# Exposure to Polybrominated Diphenyl Ethers and Phthalates in China: A Disease Burden and Cost Analysis

**DOI:** 10.3390/toxics10120766

**Published:** 2022-12-08

**Authors:** Hang Wang, Yunhui Zhang

**Affiliations:** 1 Key Lab of Health Technology Assessment, National Health Commission of the People’s Republic of China (Fudan University), Shanghai 200032, China; 2 Key Laboratory of Public Health Safety, Ministry of Educational, School of Public Health, Fudan University, Shanghai 200032, China

**Keywords:** endocrine-disrupting chemicals, China, disease burden, economic costs, Gross Domestic Product

## Abstract

Increasing evidence indicates that endocrine-disrupting chemicals (EDCs) cause a variety of adverse health outcomes and contribute to substantial disease burden. This study summarized the exposure status of polybrominated diphenyl ethers (PBDEs) and phthalates (PAEs) in China and evaluated the disease burden attributable to PBDEs and PAEs in 2015. The results showed that PBDE and PAE concentrations were higher in coastal areas. The disease burden attributable to PBDEs was 0.77 million cases, and the economic costs were CNY 18.92 billion. Meanwhile, 3.02 million individuals suffered from diseases attributable to PAEs, and the economic costs were CNY 49.20 billion. The economic burden caused by PBDEs and PAEs accounted for 0.28% and 0.72% of China’s Gross Domestic Product (GDP) in 2015, respectively. When comparing China’s results from 2010, it was determined that the GDP ratio of economic costs caused by PAEs in 2015 (0.72%) was lower than in 2010 (1.42%). Finally, compared with the results of the European Union and North America, the GDP ratios of economic costs caused by PAEs in 2015 were 0.19% in Canada (lower than China), 0.29% in the United States (lower than China), and 1.44% in the European Union (higher than China). This study provides important reference values for China’s health governance, and further research should be conducted in the future.

## 1. Introduction

Endocrine-disrupting chemicals (EDCs), known as environmental hormones, cause health effects on individuals or groups, mainly by interfering with endocrine functions. Examples of EDCs include pesticides, industrial compounds, and heavy metals [1]. Progressively more research has found that EDCs are associated with a variety of adverse health outcomes [2], such as intellectual disability, breast cancer, obesity, and diabetes [3]. In the past two decades, China has introduced numerous policies and regulations on the prevention and control of EDCs and made progress on researching, monitoring, and limiting the use of EDCs [4]. However, currently there is still insufficient understanding of the risks of EDCs. In the Global Burden of Disease Study (GBD), the only focus has been on the burden of disease attributable to lead exposure, while the adverse consequences of exposure to other EDCs have not been assessed [5].

Since the adverse effects of EDCs in humans were first identified [6], increasing evidence has supported the presumption that EDC exposure is associated with substantial disease burden. For example, several studies have reported the substantial burden of disease and economic costs due to EDC exposure in the European Union (EU). In 2014, the Health and Environment Alliance (HEAL) completed the first study on the economic costs of EDC exposure; it showed that the economic losses caused by EDCs were EUR 13–20 billion [7]. Trasande et al. reported that the economic costs attributable to EDCs in the EU in 2010 were EUR 157 billion, accounting for 1.28% of the Gross Domestic Product (GDP) that year [8]. Another study conducted in the United States (US) found that the economic costs of EDC exposure were 2.33% of GDP in 2010, much higher than they were in Europe [9]. A recent study found that the economic costs of EDC exposure in the general population in Canada were CAD 24.6 billion, much lower than those in the US (USD 340 billion) and the EU (USD 217 billion) [10]. Nevertheless, EDC exposure still causes a significant disease burden in Canada, accounting for 1.25% of Canada’s GDP [10]. China has become the world’s largest consumer of synthetic chemicals, including phthalates (PAEs) and polybrominated diphenyl ethers (PBDEs), which are widely used in industrial production [11,12] and commonly detected in the environment [13,14,15]. PAE and PBDE exposure have become a growing concern. In 2010, a study conducted by Peking University assessed the burden of disease and the economic costs attributable to PAEs among the general Chinese population [16]. After the Taiwan plasticizer incident in 2011, the use of PAEs was gradually restricted. Recently, the World Health Organization (WHO) reported that PBDEs in e-waste could affect thyroid function and impair children’s cognitive development [17], but there is still a lack of research on the disease burden caused by PBDE exposure in China. Meanwhile, with the increasing number of studies, the data conditions for assessing the disease burden caused by PBDE exposure are now available in China. This research ultimately focused on PBDEs and PAEs.

In this disease burden analysis, we assessed the burden of disease attributable to PBDEs in the general Chinese population, filling a gap in the results of related studies. Then, in light of the Taiwan plasticizer incident, this study assessed the disease burden attributable to PAEs in the general Chinese population in 2015 and compared the results with different years and other countries. Finally, this study compared the disease burden between PBDEs and PAEs and provided evidence for the prevention of priority pollutants.

## 2. Materials and Methods

### 2.1. Exposure Assessment

In this part of the study, we compiled and summarized the literature related to PBDE and PAE levels in different human tissues and fluids, including serum, cord blood, and breast milk. The literature was searched and classified through the China National Knowledge Infrastructure (CNKI, the largest Chinese database in the world), WAN-FANG DATA (a Chinese professional academic database named after CNKI), PubMed, and Web of Science between 2010 and 2020. Ultimately, 13 articles related to PBDE concentrations (Table S1) and 14 articles related to PAE concentrations (Table S2) were included in this study, and the relevant results were analyzed using ArcGIS 10.2 software.

### 2.2. Study Design

This study was designed to investigate the EDC-attributable disease burden in China. The epidemiological and toxicological evidence supported 6 exposure–response relationships between EDCs and various diseases. The EDCs evaluated were PBDEs and PAEs, and the categories for health outcomes were intellectual disability, breast cancer, thyroid cancer, adult diabetes, adult obesity, and male infertility [8,18,19,20,21,22].

We applied a model first used by the Institute of Medicine, which remains widely used to this day and is presented in Equations (1) and (2), to estimate the disease burden and costs attributable to EDC exposure [23]:

Attributable disease burden = disease rate × attributable fraction × population size(1)

Attributable cost = disease rate × attributable fraction × population × cost per case(2)

An attributable fraction (AF) refers to the “proportional reduction in average disease risk over a specified time interval that would be achieved by eliminating the exposure(s) of interest from the population while distribution of other risk factors in the population remained unchanged [24]” and can be estimated by the following equation:

(3) $$\mathrm{Attributable}\;\mathrm{fraction}=\frac{{\mathrm{prevalence}}_{\mathrm{exposure}}\times (\mathrm{RR}-1)}{{1+(\mathrm{prevalence}}_{\mathrm{exposure}}\times \left(\mathrm{RR}-1\right))}$$

Note: The AF is determined using (i) the prevalence of exposure (Pe), i.e., the exposed proportion of the entire population, and (ii) relative risk (RR), or conversion from the corresponding odds ratio (OR) obtained from epidemiological studies [23].

### 2.3. Data Collection

Data for the AFs of the target diseases caused by PBDEs were calculated using Equation 3 (Table S3), and the AFs of the target diseases caused by PAEs were calculated by Peking University in 2010 (Table S4) [16]. This assessment was based on the premise that the PAE levels and OR values for the target diseases were comparable between the general Chinese and European populations (Tables S5–S7), and the AFs of the general Chinese population in 2015 were comparable to those of the general Chinese population in 2010 (Table S8). The Pe of PBDEs, the OR values of the target diseases caused by PBDEs, and the prevalence rates of the target diseases were obtained through meta-analysis (if only one study was available, the present study used its research data directly). The literature was searched and classified through CNKI, WAN-FANG DATA, PubMed, and Web of Science, and the PRISMA guidelines were followed. Figures S1 and S2 depict the specific literature retrieval process. Pe could not be obtained directly from the literature, so we included 11 papers about PBDEs’ detection rate and used the meta-analysis results for detection rate to estimate Pe (Table S9). The ORs for thyroid cancer and breast cancer were derived from relevant studies in China [18,21,22]. For intellectual disability, due to a lack of corresponding data in China, data from Europe and the US at the same exposure levels were used for estimation [19,20]. The literature on the prevalence rate of the target diseases is detailed in Tables S10–S15. It should be noted that the prevalence rate is somewhat time-dependent, so this study collected the literature about prevalence rates between 2011 and 2020 to ensure that the derived prevalence rate was representative of the situation around 2015. A uniformly designed Excel form was used for data extraction, and the extracted content contained basic information from the literature. A meta-analysis was performed using the meta package (R3.6.2). Heterogeneity testing revealed that the detection rate for PBDEs, the ORs of the target diseases caused by PBDEs, and the prevalence rates of the target diseases in different studies were heterogeneous, so a random effects model was used to combine them in the meta-analysis (*p* < 0.05).

### 2.4. Estimates of Economic Costs

The economic costs for each disease were calculated by applying a cost-of-illness approach to direct costs, indirect costs, and intangible costs. The cost per case was summarized and combined using the meta-analysis (Figure S3, Tables S16–S19). Given that the estimates spanned more than a decade from 2006 to 2019, in order to make these results comparable, the Consumer Price Index (CPI) was used to uniformly adjust them to 2015 prices (data from China’s National Bureau of Statistics). Figure S4 shows the data input/output process.

### 2.5. Statistical Analysis

ArcGIS 10.2 was used to assess the spatial and temporal distribution of PBDE and PAE exposure in the general population of China, and R3.6.2 was used to conduct the meta-analysis.

## 3. Results

### 3.1. Spatial and Temproal Distribution of PBDE and PAE Levels in the General Chinese Population

**Figure 1:**
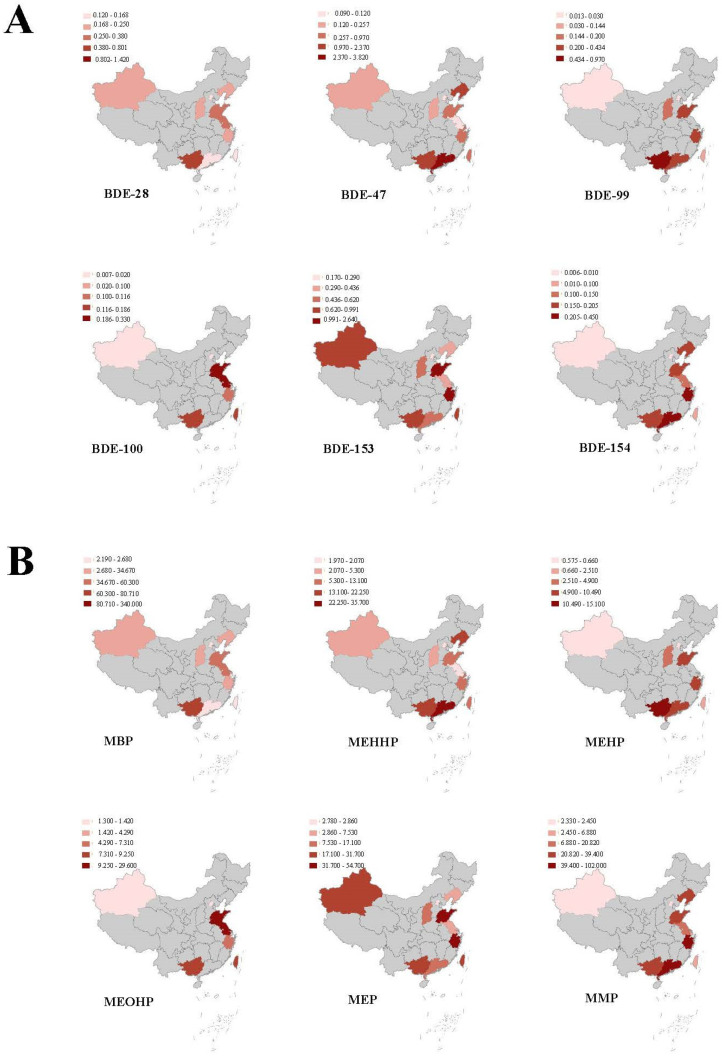
(**A**) Spatial and temporal distribution of PBDE levels in the general Chinese population (ng/g lipid). (**B**) Spatial and temporal distribution of PAE levels in the general Chinese population (μg/L). Note: The darker the area, the higher the exposure levels (median exposure concentration of PBDEs and PAEs in this area). The regions with gray marks had no data. (China, 2011–2020). Abbreviations: PBDEs, polybrominated diphenyl ethers; PAEs, phthalates.

The results showed that studies on PBDE levels were mainly concentrated in the coastal areas and a few inland areas of China (Figure 1A). People in the Guangxi, Guangdong, Shandong, and Zhejiang provinces of China were exposed to higher PBDE concentrations than elsewhere. The total concentrations of PBDEs in the serum of Laizhou Bay residents in Shandong Province were the highest, reaching 64.5 ng/g lipid, and the average concentration of PBDEs in the breast milk of lactating women in Beijing was 3.24 ng/g lipid.

Similarly, China’s PAE-related exposure assessment studies were mostly concentrated in the eastern region (Figure 1B). Among them, the Northeast, Jiangsu Province, Zhejiang Province, and the Pearl River Delta region had higher PAE concentrations. The average concentrations of PAEs in the urine of the general population in Guangdong Province were as high as 552 μg/L.

### 3.2. Attributable Disease Burden Caused by PBDEs and PAEs in China

Table 1 shows the estimated disease burden caused by PBDEs and PAEs in the general Chinese population in 2015. The number of PBDE-related disease cases was 0.77 million, with thyroid cancer having the highest number of cases among the three PBDE-related target diseases.

In 2015, 3.02 million individuals suffered from PAE-related diseases; the highest number of cases was male infertility, followed by diabetes and adult obesity.

### 3.3. Estimate of Costs for Disorders Associated with PBDE and PAE Exposure in China

Table 2 shows the economic costs of the target diseases attributable to PBDEs and PAEs. The economic costs of thyroid cancer associated with PBDEs were CNY 15.38 billion, while PBDE-related breast cancer resulted in economic losses of CNY 3.23 billion. The economic costs of intellectual disability were the lowest among the three PBDE target diseases at approximately CNY 310 million. Overall, the total economic costs were about CNY 18.92 billion. The economic burden caused by PBDEs accounted for 0.28% of GDP in 2015.

In 2015, the health costs of the target diseases caused by PAEs were CNY 49.20 billion. The economic costs of male infertility were CNY 31.46 billion, while the economic costs of diabetes were CNY 16.01 billion, and those of adult obesity were CNY 1.73 billion. The economic burden of diseases attributable to PAEs as a share of GDP was 0.72%.

### 3.4. Comparison of Disease Burden Attributable to PAEs between Different Years

In this study, the results of the evaluations in 2010 and 2015 were compared, mainly through case numbers, economic costs, and GDP ratios (Table 3). First, the number of disease cases caused by PAEs was 2.54 million in 2010, which was less than the number of disease cases (3.02 million) determined by this study (2015). As for economic costs, the evaluation results in 2010 showed that the economic losses caused by PAE exposure were CNY 57.20 billion, which were higher than those found in this study (CNY 49.20 billion). Finally, the total economic costs attributable to PAEs accounted for 0.72% of GDP in 2015, which was lower than it was in 2010 (1.42%).

### 3.5. Comparison of Attributable Disease Burdens between Different Countries

In order to compare the disease burden between different countries, this study calculated the economic costs of EDC exposure as a share of GDP, which was a reasonable approach for comparing the impact of disease burden on the national economies of different countries without having to account for currency exchange rates. For intellectual disability attributable to PBDEs, the number of disease cases was similar between China and the US, but far higher than the number of cases in the EU and Canada. China’s economic costs in terms of impact on GDP were far lower than the economic costs in the US, the EU, and Canada (Table 4).

As for PAEs, the number of cases in China far exceeded the number of cases in the US, the EU, and Canada (Table 4). The total costs of diseases attributable to PAE exposure accounted for 0.19% of GDP in Canada (lower than China), 0.29% of GDP in the US (lower than China), and 1.44% of GDP in the EU (higher than China).

## 4. Discussion

In this study, we estimated the disease burden and economic costs attributable to PBDEs and PAEs. First, it was determined that the number of disease cases attributable to PBDEs was 0.77 million, and the economic costs were CNY 18.92 billion. When compared with results from other countries, the number of intellectual disability cases in China was similar to the number in the US, but far higher than the number in the EU and Canada. However, the impact of the economic costs of intellectual disability on GDP was far lower in China than in the US, the EU, and Canada. PAE-related diseases affected 3.02 million individuals, and the healthcare costs attributable to PAEs were CNY 49.20 billion. Compared with the results for China in 2010, there were more disease cases in 2015, but the economic costs were lower than they were in 2010. When comparing China with other countries, we found that the total costs of disease attributable to PAE exposure accounted for 0.19% of GDP in Canada (lower than China), 0.29% of GDP in the US (lower than China), and 1.44% of GDP in the EU (higher than China). Finally, when comparing the disease burden caused by PBDEs and PAEs in this study, it was found that the disease burden and economic costs associated with PAEs were substantial, which could provide a basis for the prevention and control of key pollutants.

This study summarized the literature on exposure concentrations of PBDEs and PAEs. At present, human exposure data are limited, so they need to be supplemented in the future. However, based on the available data, several important exposure–response relationships could be established for subsequent disease burden analysis.

Current studies on the disease burden associated with PBDEs have mainly focused on neurotoxicity. Gaylord et al. reported that the economic costs of IQ loss and intellectual disability attributable to PBDEs were USD 4551.65 billion from 2001 to 2016 [25]. Another study conducted by the EU found that the economic costs of intellectual disability caused by PBDEs in 2010 were EUR 9.59 billion [8]. These results reflect the substantial disease burden caused by PBDEs, which are consistent with our findings. This study also focused on breast cancer and thyroid cancer, which fills the gap in related research and has considerable public health significance.

In this study, the disease burden caused by PAEs was compared in time and space. First, a study conducted by Peking University assessed the disease burden and economic costs associated with PAE exposure in the general Chinese population in 2010 [16]. The results showed that the costs of disease caused by PAE exposure were as high as CNY 57.20 billion, accounting for 1.42% of GDP in 2010, which was higher than it was in 2015. The reasons for the difference between 2010 and 2015 are as follows: after the Taiwan plasticizer incident in 2011, the EU issued a related ban on phthalic plasticizers [26], and the use of phthalic plasticizers in the Chinese market was also restricted.

The economic costs attributable to PAE exposure in the general Chinese population in 2015 were higher than in the US and Canada but lower than in the EU [8,9]. These differences may be related to differences in policies between China, the EU, and North America. The EU, North America, and other developed countries have attached great importance to the control of EDCs since the 1990s and formulated relevant management regulations and strategic control plans. For example, the US has established a two-level screening and testing program and has included a variety of EDCs on its priority pollutant control list [27]. Similar to the US, Canada used a risk-based strategy for the regulation of chemicals under the Canadian Environmental Protection Act of 1999 [28]. As for the EU, the disease burden due to PAE exposure in the general Chinese population in 2010 was comparable to that in the EU (1.42% vs. 1.44%) [8,16]. However, the disease burden due to PAE exposure in the general Chinese population in 2015 was lower than it was in the EU, which may be explained by the restrictions placed on the use of plasticizers following the Taiwan plasticizer incident.

This study mainly focused on PBDEs and PAEs. However, organophosphate esters (OPEs), bisphenol A (BPA), dichlorodiphenyltrichloroethane (DDT), and heavy metals have also been reported to account for a proportion of the disease burden, such as intellectual disability, childhood obesity, adult diabetes, and female uterine fibroids. For example, a study in the US found that OPE exposure caused economic losses of USD 593.67 billion between 2001 and 2016 [25]. In 2010, the economic costs of childhood obesity caused by BPA were USD 2.4 billion in the US [9] and EUR 1.54 billion in the EU [8]. However, there is still a lack of relevant studies in China, and further attention should be paid to common EDCs for a comprehensive disease burden assessment.

China has become the world’s largest consumer of synthetic chemicals. The general population is widely exposed to EDCs, which fully reflects the necessity of assessing the disease burden attributable to EDCs in China. Our research is forward-looking and will help fill the gaps in this type of research; it will also provide important reference values for China’s health governance. This study evaluated the disease burden of breast cancer and thyroid cancer attributable to PBDEs. It provides a scientific basis for the implementation of health policies and protection of the population’s health to a great extent.

It must be acknowledged that the costs estimated in this study represent only a fraction of the total costs of exposure to PBDEs and PAEs in the general Chinese population. First, China lacked data on the indirect economic burden of some diseases, such as intellectual disability and thyroid cancer. For example, thyroid cancer is a chronic disease that has a long course of disease and is life-threatening, so its indirect economic burden can be very high. Second, in view of the current relevant research basis, our study only evaluated six exposure–response relationships. However, PBDE and PAE exposure can increase the risk of many other diseases. For example, many recent studies have shown associations between PBDEs and PAEs with pediatric and adult liver diseases, particularly non-alcoholic fatty liver disease (NAFLD) [29]. Future studies should add to the assessment of the corresponding disease burden. This study evaluated a small subset of EDCs that had to have sufficiently robust exposure, toxicological, and epidemiological evidence to be eligible for inclusion in the analysis [30]. However, other EDCs may lead to a variety of diseases, which can also cause a substantial disease burden. Ultimately, this study focused on assessing the effects of exposure to two individual chemicals rather than combined exposure to EDCs, so further exploration of how different EDCs collectively cause diseases should be undertaken [31].

The results of this study may be subject to uncertainty. First, with time changes, every link of the disease burden constantly changes. The results of the study are an estimate obtained after comprehensive consideration of various factors. Second, the AFs of some diseases in this study were calculated directly. The two required parameters were odds ratio and prevalence of exposure. Because some diseases lacked relevant data in China, European and US data were used instead. Therefore, the AF calculations were based on the assumption that these data could represent the general Chinese population.

## 5. Conclusions

This study performed an analysis of the disease burden attributable to EDC exposure in China, with the following key conclusions:

(1) The number of disease cases attributable to PBDEs was 0.77 million, and the economic costs were CNY 18.92 billion. The number of intellectual disability cases was similar to that of the US, but far higher than the number in the EU and Canada; however, the economic costs they accounted for in China’s GDP were far lower than those in the US, the EU, and Canada.
(2) A total of 3.02 million individuals suffered from PAE-related diseases, and the healthcare costs attributable to PAEs were CNY 49.20 billion. There were more disease cases in 2015 than in 2010, but the economic costs were lower in 2015 than in 2010. When compared with other countries, we found that the total costs of disease attributable to PAE exposure accounted for 0.19% of GDP in Canada (lower than China), 0.29% of GDP in the US (lower than China), and 1.44% of GDP in the EU (higher than China).
(3) The disease burden caused by PAEs was more substantial than PBDEs, which could provide a basis for the prevention and control of key pollutants.

## Figures and Tables

**Table 1:** Attributable disease burden caused by PBDEs and PAEs in the general Chinese population in 2015.

EDCs	Health Outcomes	Target Population	Base Case (Ten Thousand)	Low Case	High Case
PBDEs	Intellectual disability	All neonates	5.2	4.4	6.2
	Breast cancer	All women	11.8	8.8	15.8
	Thyroid cancer	All adults	60.0	17.6	204.7
PAEs	Diabetes	Adults aged 40–59 years	89.9	75.5	104.3
	Adult obesity	Adults aged 40–59 years	83.7	72.5	94.8
	Male infertility	Men aged 20–39 years	128.9	76.4	183.7

Abbreviations: EDCs, endocrine-disrupting chemicals; PBDEs, polybrominated diphenyl ethers; PAEs, phthalates.

**Table 2:** Estimate of costs for disorders associated with PBDE and PAE exposure in the general Chinese population (2015).

Exposure–Response Relationship	Base Estimate (Billion CNY)	Low Estimate	High Estimate
PBDE and intellectual disability			
Direct disease cost	0.31	0.26	0.37
Indirect disease cost	-	-	-
Intangible disease cost	-	-	-
Total disease cost	0.31	0.26	0.37
PBDE and breast cancer			
Direct disease cost	2.24	1.67	3.00
Indirect disease cost	0.99	0.74	1.33
Intangible disease cost	-	-	-
Total disease cost	3.23	2.41	4.33
PBDE and thyroid cancer			
Direct disease cost	15.38	4.51	52.50
Indirect disease cost	-	-	-
Intangible disease cost	-	-	-
Total disease cost	15.38	4.51	52.50
PAE and diabetes			
Direct disease cost	7.10	5.96	8.24
Indirect disease cost	4.08	3.42	4.73
Intangible disease cost	4.83	4.06	5.61
Total disease cost	16.01	13.44	18.58
PAE and adult obesity			
Direct disease cost	0.84	0.72	0.95
Indirect disease cost	0.90	0.78	1.02
Intangible disease cost	-	-	-
Total disease cost	1.73	1.50	1.97
PAE and male infertility			
Direct disease cost	30.59	18.14	43.61
Indirect disease cost	0.50	0.30	0.71
Intangible disease cost	0.36	0.22	0.52
Total disease cost	31.46	18.66	44.84

Abbreviations: PBDEs, polybrominated diphenyl ethers; PAEs, phthalates.

**Table 3:** Comparison of attributable disease burdens in the general Chinese population in 2010 and 2015.

Exposure–Response Relationship	2010		2015	
	Disease Burden (Thousand)	Economic Costs (Billion CNY)	Disease Burden (Thousand)	Economic Costs (Billion CNY)
PAE and diabetes	676.1	10.92	899.0	16.01
PAE and adult obesity	820.1	21.66	837.0	1.73
PAE and male infertility	1043.2	24.62	1289.0	31.46

Abbreviation: PAEs, phthalates.

**Table 4:** Comparison of the attributable disease burden between China (2015) and the US, the EU, and Canada (2010).

Exposure–Response Relationship	US	EU	Canada	China
	Disease Burden			
PBDE and intellectual disability	43,000 cases	3290 cases	1610 cases	52,000 cases
PBDE (account for GDP)	17.98%	0.65%	0.47%	0.005%
PAE and diabetes	1300 cases	20,500 cases	225 cases	899,000 cases
PAE and adult obesity	5900 cases	53,900 cases	2093 cases	837,000 cases
PAE and male infertility	240,100 cases	618,000 cases	1395 cases	1,289,000 cases
Total (account for GDP)	0.29%	1.44%	0.19%	0.72%

Abbreviations: PBDEs, polybrominated diphenyl ethers; PAEs, phthalates; GDP, Gross Domestic Product.

## Data Availability

Data regarding CPI were obtained from China’s National Bureau of Statistics.

## Supplementary Materials

The following supporting information can be downloaded at https://www.mdpi.com/article/10.3390/toxics10120766/s1; Figures S1–S4, and Tables S1–S19 are in the Supplementary Materials. References [32,33,34,35,36,37,38,39,40,41,42,43,44,45,46,47,48,49,50,51,52,53,54,55,56,57,58,59,60,61,62,63,64,65,66,67,68,69,70,71,72,73,74,75,76,77,78,79,80,81,82,83,84,85,86,87,88,89,90,91,92,93,94,95,96,97,98,99,100,101,102,103,104,105,106,107,108,109,110,111,112,113,114,115,116,117,118,119,120,121,122,123,124,125,126,127,128,129,130,131,132,133,134,135,136,137,138,139,140,141,142,143,144,145,146,147,148,149,150,151,152,153,154,155,156,157,158,159,160,161,162,163,164,165,166,167,168,169,170,171,172,173,174,175,176,177,178,179,180,181,182,183,184,185,186,187,188,189,190,191,192,193,194,195,196,197,198,199,200,201,202,203,204,205,206,207,208,209,210,211,212,213,214,215,216,217,218,219,220,221,222,223,224,225,226,227,228,229,230,231,232] is in the Supplementary Materials.
