# Supplementary material for: Exposure to Polybrominated Diphenyl Ethers and Phthalates in China: A Disease Burden and Cost Analysis
Source: Toxics. 2022 Dec 8;10(12):766. doi: 10.3390/toxics10120766 (PMC9782749; doi:10.3390/toxics10120766)
Supplement: Supplementary file 1 [file toxics-10-00766-s001.zip › toxics-2011338-supplementary.pdf]

Supplementary Materials

Exposure to polybrominated diphenyl ethers and phthalates in China: a disease burden and cost analysis

Hang Wang [1‡], Yunhui Zhang [2,*]

[1]Key Lab of Health Technology Assessment, National Health Commission of the People's Republic of China (Fudan University), China; Key Laboratory of Public Health Safety, Ministry of Educational, School of Public Health, Fudan University, Shanghai 200032, China; 20111020036@fudan.edu.cn

[2] Key Lab of Health Technology Assessment, National Health Commission of the People's Republic of China (Fudan University), China; Key Laboratory of Public Health Safety, Ministry of Educational, School of Public Health, Fudan University, Shanghai 200032, China; yhzhang@shmu.edu.cn

*Corresponding authors: Yunhui Zhang, School of Public Health, Fudan University, 138 Yixueyuan Road, Shanghai 200032, China. Phone: +86-21-54237085. Email: yhzhang@shmu.edu.cn.

Number of pages: 30

Number of figures: 4

Number of tables: 19

Table of Contents Page

Figure S1. Flow chart summarizing the detection rate related study selection procedure. 3
Figure S2. Flow chart summarizing the prevalence rate related study selection procedure. 4
Figure S3. Flow chart summarizing the disease cost related study selection Procedure. 5
Figure S4. The diagram outlining the data input/output process. 6
Table S1. Related researches on the concentrations of PBDEs in Chinese population. 7
Table S2. Related researches on the concentrations of PAEs in Chinese population. 7
Table S3. Attributable fraction of polybrominated diphenyl ethers-attributable diseases. 8
Table S4. Attributable fraction of phthalates-attributable diseases derived from the EU study. 8
Table S5. Comparison of OR in China and western countries (adult obesity). 8
Table S6. Comparison of OR in China and western countries(diabetes). 9
Table S7. Comparison of OR in China and western countries (male infertility). 10
Table S8. Comparison of OR between 2010 and 2015. 10
Table S9. Related researches on the detection rate of PBDEs in Chinese population. 11
Table S10. The prevalence studies of prevalence rate of intellectual disability in China. 11
Table S11. The prevalence studies of prevalence rate of breast cancer in China. 12
Table S12. The prevalence studies of prevalence rate of thyroid cancer in China. 13
Table S13. The prevalence studies of prevalence rate of adult obesity in China. 14
Table S14. The prevalence studies of prevalence rate of diabetes in China. 15
Table S15. The prevalence studies of prevalence rate of male infertility in China. 15
Table S16. Disease cost of intellectual disability in China. 15
Table S17. Disease cost of breast cancer in China. 16
Table S18. Disease cost of thyroid cancer in China. 16
Table S19. Disease cost of diabetes, adult obesity and male infertility in China. 16

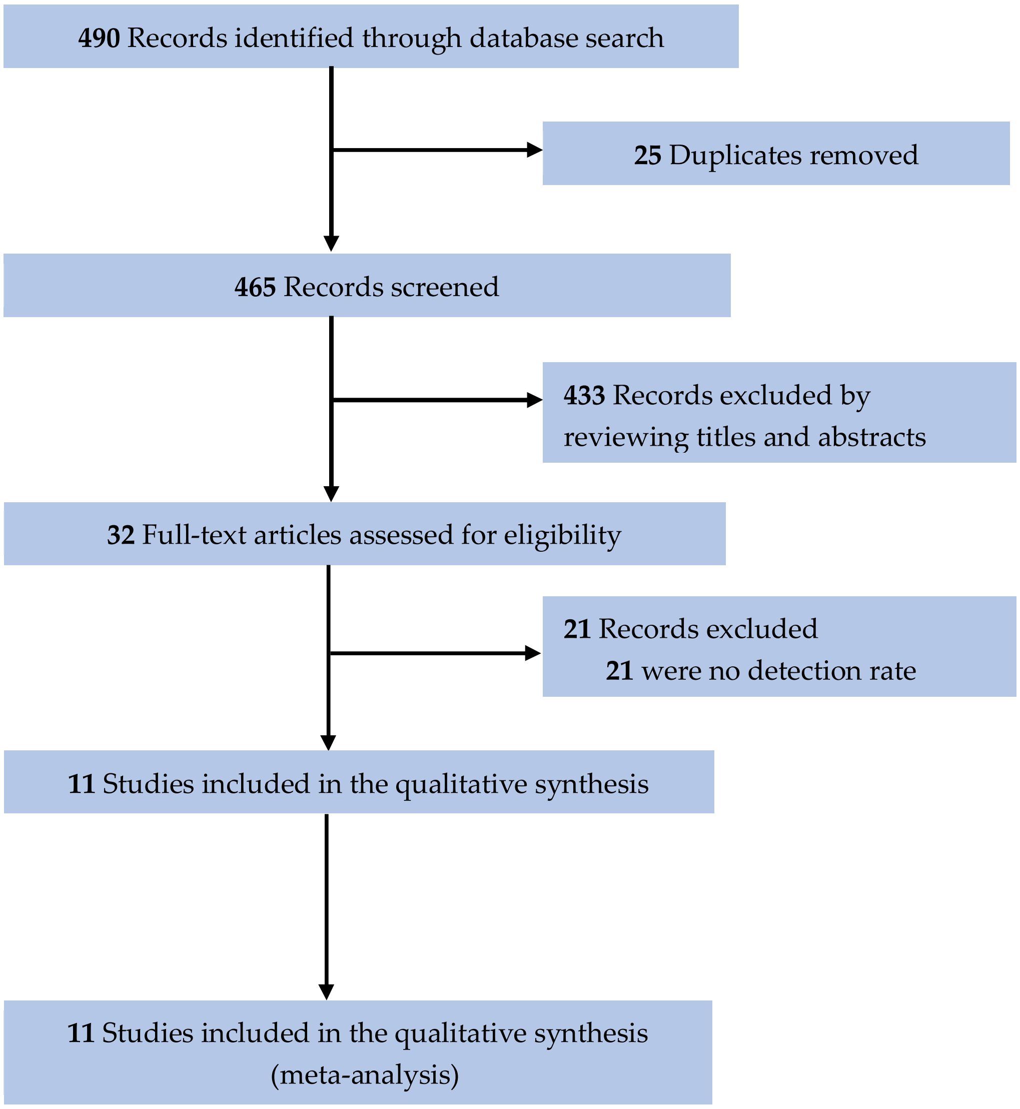

Figure S1. Flow chart summarizing the detection rate related study selection procedure.

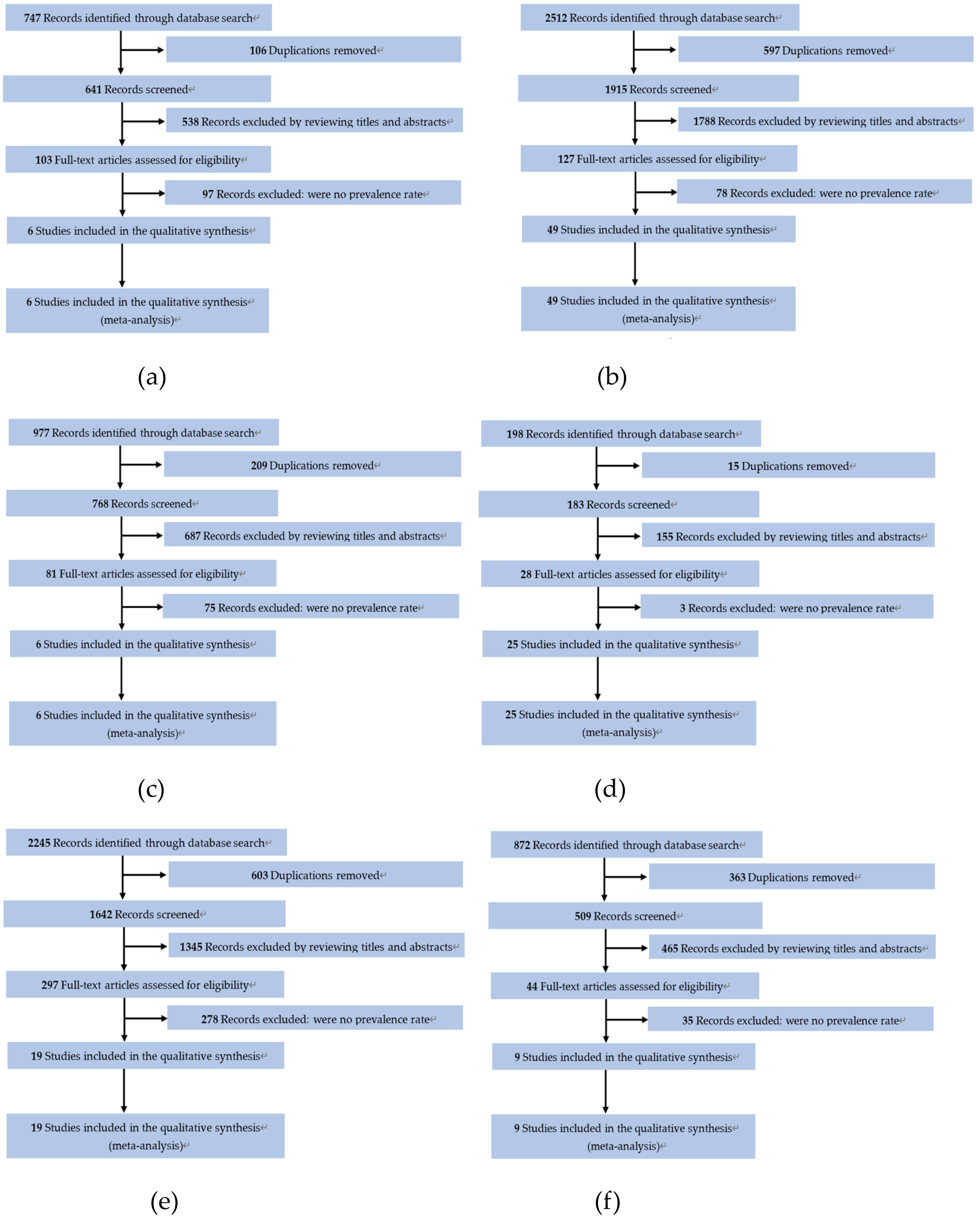

Figure S2. Flow chart summarizing the prevalence rate related study selection procedure. Note: **(a)** intellectual disability; **(b)** breast cancer; **(c)** thyroid cancer; **(d)** adult obesity; **(e)** diabetes; **(f)** male infertility.

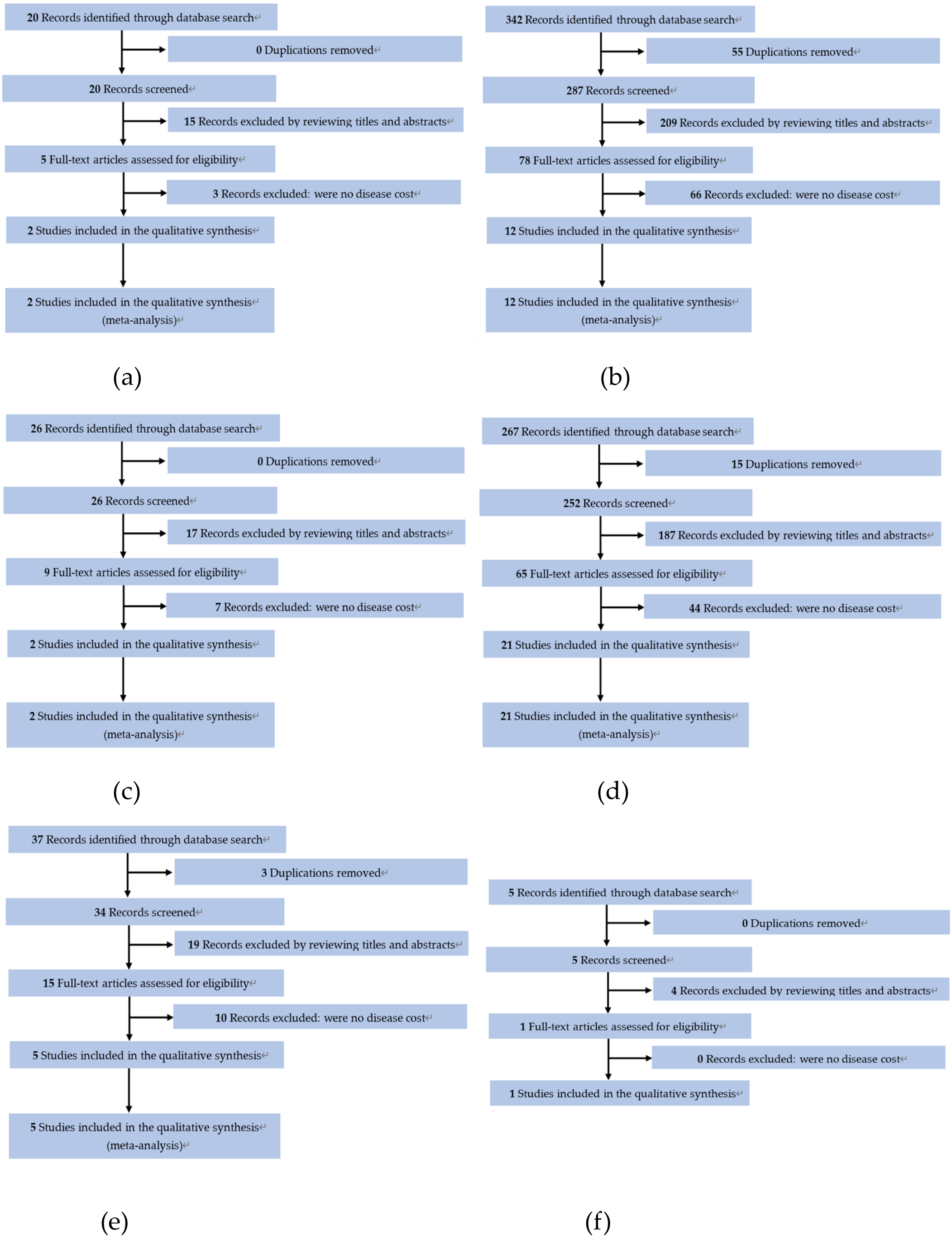

Figure S3. Flow chart summarizing the disease cost related study selection procedure. Note: **(a)** intellectual disability; **(b)** breast cancer; **(c)** thyroid cancer; **(d)** adult obesity; **(e)** diabetes; **(f)** male infertility.

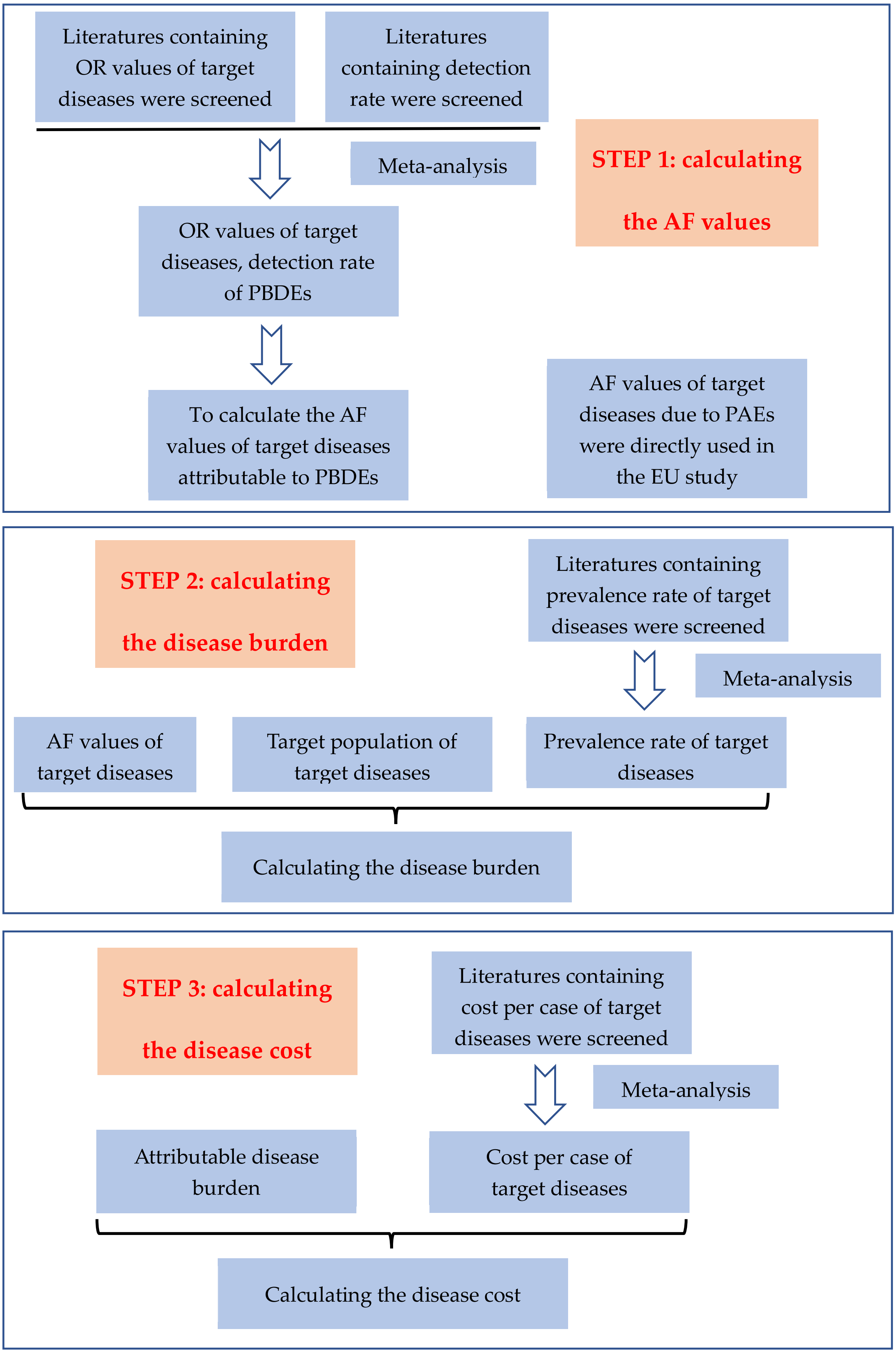

Figure S4. The diagram outlining the data input/output process.

Table S1. Related researches on the concentrations of PBDEs in Chinese population.

Year of publication	Area	Crowd	Sample Size	Sample type	References
2020	Shandong	resident	172	serum	[32]
2020	Xinjiang	pregnant women	96	serum	[33]
2020	Jiangsu	mother and child	318	cord blood	[34]
2018	Shanghai	resident	25	serum	[35]
2016	Liaoning	volunteer	32	serum	[36]
2016	Hebei	volunteer	43	breast milk	[37]
2015	Zhejiang	pregnant women	64	serum	[38]
2014	Shanxi	volunteer	124	serum	[39]
2014	Guangdong	mother and child	30	cord blood	[40]
2013	Beijing	volunteer	103	breast milk	[41]
2013	Hong Kong	volunteer	117	serum	[42]
2011	Taiwan	pregnant women	70	breast milk	[43]
2010	Guangxi	pregnant women	102	cord blood	[44]

Abbreviations: PBDEs, polybrominated diphenyl ethers.

Table S2. Related researches on the concentrations of PAEs in Chinese population.

Year of publication	Area	Crowd	Sample Size	Sample type	References
2020	Guangdong	general population	360	urine	[45]
2019	Shanghai	children	154	urine	[46]
2019	Hubei	pregnant women	947	urine	[47]
2019	Chongqing	children	210	urine	[48]
2017	Anhui	pregnant women	2838	urine	[49]
2016	Heilongjiang	college students	366	serum	[50]
2015	Jilin	teenagers	25	urine	[51]
2015	Liaoning	teenagers	25	urine	[51]
2015	Hunan	children	216	serum	[52]
2013	Jiangsu	children	968	urine	[53]
2013	Zhejiang	children	968	urine	[53]
2011	Ningxia	women	30	serum	[54]
2020	Beijing	newborns	161	cord blood	[55]
2019	Guangxi	ethnic minorities	950	serum	[56]

Abbreviations: PAEs, phthalates.

Table S3. Attributable fraction of polybrominated diphenyl ethers-attributable diseases (2015, China).

Target diseases	AFs (in %)
Intellectual disability	9.0
Breast cancer	44.5
Thyroid cancer	67.9

Abbreviations: AF, attributable fraction.

Table S4. Attributable fraction of phthalates-attributable diseases derived from the EU study (2015, China).

Target diseases	AFs (in %)
Adult obesity	0.65
Diabetes	0.64
Male infertility	6.43

Abbreviations: EU, European Union; AF, attributable fraction.

Table S5. Comparison of OR in China and western countries (adult obesity).

Research/year of publication	substance	Country/ region	crowd	Sample size	Exposure level	OR(95%CI)
Yaghjyan /2015[57]	MBP	America	adult women	1702	22.14μg/g Cr	1.13 (1.03, 1.23)
Li/2020[58]	MBP	Anhui	old people	942	44.08~79.8μg/L	2.78 (1.84, 4.21)
Hou/2015 [59]	MiBP	Taiwan	teenager	270	31.14~46.67μg/L	1.72 (0.73, 4.03)
Buser/2014 [60]	MiBP	America	teenager	/	5.38~20.84μg/L	1.82 (0.73, 4.57)
Zhang/2014 [61]	∑LMP	Shanghai	school-age boy	247	37.4~282.7μg/L	6.841(2.073,22.57)
Buser/2014 [60]	∑LMP	America	male adolescent	/	0.26~0.10μmol/mL	5.39(1.87,15.53)
Buser/2014 [60]	MBzP	America	teenager	/	5.66~27.58μg/L	2.15 (0.80, 5.57)
Hou/2015 [59]	MBzP	Taiwan	teenager	270	3.97~8.3μg/L	1.13(0.48, 2.65)
Yaghjyan /2015[57]	MEHP	America	adult women	1702	3.07μg/g Cr	1.12 (1.03, 1.23)
Li/2020[58]	MEHP	Anhui	old people	942	0.55~22.42μg/L	1.50 (1.02, 2.22)

Abbreviations: OR, odd ratio.

Table S6. Comparison of OR in China and western countries(diabetes).

Research/year of publication	substance	Country/region	crowd	Sample size	Exposure level	OR(95%CI)
Dong/2017[62]	MnBP	Shanghai	resident(≥18y)	3322	12.92~37.10μg/g Cr	1.19 (0.74, 1.93)
Huang/2014[63]	MnBP	America	resident(12-80y)	3083	22.3~35.9μg/g Cr	1.41 (0.72, 2.09)
Dong/2017[62]	MiBP	Shanghai	resident(≥18y)	3322	8.42~19.38μg/g Cr	1.32 (0.81, 2.17)
Duan/2019[64]	MiBP	Tianjin	outpatient	500	9.11~20.7μg/L	1.06 (0.55, 2.05)
Lind/2012[65]	MiBP	Sweden	resident(70y)	1016	9.3~13.5 μg/L	1.19 (0.59, 2.38)
Dong/2017[62]	MiBP	Shanghai	Resident(≥18y)	3322	1.91~8.42μg/g Cr	1.18 (0.74, 1.91)
Huang/2014[63]	MiBP	America	resident(12-80y)	3083	4.9~8.9μg/g Cr	1.23 (0.57, 1.89)
Dong/2017[62]	MBzP	Shanghai	resident(≥18y)	3322	4.10~22.36 μg/g Cr	1.06 (0.66, 1.69)
Huang/2014[63]	MBzP	America	resident(12-80y)	3083	13.4~23.8μg/g Cr	1.09 (0.39, 1.79)
Dong/2017[62]	MEHP	Shanghai	resident	3322	8.84~70.95μg/g Cr	1.10 (0.69, 1.77)
Lind/2012[65]	MEHP	Sweden	resident(70y)	1016	4.5~15.5 μg/L	1.61 (0.81, 3.2)
Duan/2019[64]	MEP	Tianjin	outpatient	500	11.12~30.64μg/L	1.50 (0.75, 2.97)
Lind/2012[65]	MEP	Sweden	resident(70y)	1016	11.6~17.5μg/L	2.87 (1.37, 6.03)

Abbreviations: OR, odd ratio.

Table S7. Comparison of OR in China and western countries (male infertility).

research/year of publication	substance	Outcome (sperm)	country/ region	sample size	exposure level	OR(95%CI)
Han/2014 [66]	MBP	initiative	Chongqing	232	23.26~157.33μg/g Cr	1.08 (0.69, 1.69）
Hauser/2006 [67]	MBP	initiative	Boston	463	17.7~31.7μg/L	1.5 (0.8, 2.6)
Han/2014 [66]	MBP	concentration	Chongqing	232	23.26~157.33μg/g Cr	1.97 (0.97, 4.0）
Hauser/2006 [67]	MBP	concentration	Boston	463	31.7~69.9μg/L	3.3 (1.2, 8.5)
Wang/2015 [68]	MBP	concentration	Wuhan	1040	68.23~133.08μg/g Cr	2.01 (1.1, 3.8)
Hauser/2006 [67]	MEOHP	concentration	Boston	463	32.1~73μg/L	1.1 (0.3, 4.6)
Liu/2012[69]	MEOHP	concentration	Chongqing	125	1.89~2.25μg/g Cr	1.4 (0.3, 7.7)
Wang/2015 [68]	MEP	concentration	Wuhan	1040	13.94~26.21μg/g Cr	1.08 (0.58, 2.04)
Hauser2006 [67]	MEP	concentration	Boston	463	58.7~157.9μg/L	1.5 (0.7, 3.6)
Liu 2012[69]	MEP	concentration	Chongqing	125	15.2~28.5μg/g Cr	1.5 (0.2, 9.6)
Hauser2006 [67]	MBzP	initiative	Boston	463	8~15.5μg/L	1.3 (0.8, 2.3)
Wang2015 [68]	MBzP	initiative	Wuhan	1040	2.13~3.93μg/g Cr	1.05 (0.73, 1.52)

Abbreviations: OR, odd ratio.

Table S8. Comparison of OR between 2010 and 2015.

Year of study	Region	Sample size	OR (95%CI)	References
**adult obesity**				
2010	Shanghai	493	2.12 (0.71, 6.35)	[61]
2015	Taiwan	270	1.72 (0.73, 4.03)	[59]
**diabetes**				
2010	Tianjin	500	1.50 (0.75, 2.97)	[64]
2015	Shanghai	574	1.10 (1.07, 1.13)	[70]
**male infertility**				
2010	Chongqing	150	1.00 (0.40, 3.10)	[69]
2015	Wuhan	1040	1.08 (0.58, 2.04)	[68]

Table S9. Related researches on the detection rate of PBDEs in Chinese population.

Year of publication	Area	Crowd	Sample size	Detection rate(%)	References
2021	Shanghai	children	281	98.58（96.40~99.61）	[71]
2020	Shandong	pregnant women	121	100.00（97.00~100.00）	[72]
2020	Shandong	worker	30	100.00（88.43~100.00）	[73]
2019	Shanghai	baby boy	190	97.89（94.70~99.42）	[74]
2019	Shanghai	newborn	340	98.82（97.02~99.68）	[75]
2019	Shanghai	children	340	98.63（96.60~99.52）	[76]
2019	Beijing	pregnant women	231	100.00（98.42~100.00）	[77]
2019	Shangdong	resident	1808	100.00（99.80~100.00）	[78]
2018	Wenzhou	newborn	121	94.21（88.44~97.64）	[79]
2012	Shanghai	parturient	48	100.00（92.60~100.00）	[80]
2011	Weifang	resident	106	100.00（96.58~100.00）	[81]

Abbreviations: PBDEs, polybrominated diphenyl ethers.

Table S10. The prevalence studies of prevalence rate of intellectual disability in China.

Year of publication	Region	Age	Sample size	Prevalence rate(‰)	Reference
2017	China	children(0-17y)	616940	9.06（8.82~9.30）	[82]
2014	Yangzhou	children(0-6y)	72036	1.85（1.55~2.19）	[83]
2012	Henan	women	58618	4.40（3.87~4.95）	[84]
2011	China	resident	1317333333	7.50（7.49~7.51）	[85]
2011	Zhejiang	resident	46773399	4.06（4.04~4.08）	[86]
2011	China	minority	297761	3.80（3.70~3.90）	[87]
		Han nationality	2228376	4.40（4.30~4.40）	

Table S11. The prevalence studies of prevalence rate of breast cancer in China.

Year of publication	Region	Age	Sample size	Prevalence rate(100000)	Reference
2019	Lanzhou	women(20-64y)	482759	26.93（22.50~31.97）	[88]
2019	Shanghai	women(20-65y)	117503	25.53（17.23~36.45）	[89]
2019	Dalian	women(25-70y)	46846	38.42（22.77~60.72）	[90]
2019	Shanghai	married women	200262	20.00（14.27~27.20）	[91]
2017	Zhengzhou	women(18-80y)	6310	60.00（17.27~162.22）	[92]
2017	Yibin	women(35-64y)	15329	13.00（1.58~47.12）	[93]
2017	Yili	women(22-99y)	4441	490.00（310.71~ 749.06）	[94]
2017	Weihai	women(30-60y)	6000	50.00（10.31~146.05）	[95]
2016	China	women	175000000	156.00（138.05~175.63）	[96]
2016	Eerduosi	women(≥20y)	36811	24.45（11.18~46.61）	[97]
2016	Yizheng	women(35-69y)	25054	60.00（36.51~103.69）	[98]
2016	Jingjiang	women(35-64y)	81642	35.52（23.79~51.01）	[99]
2016	Xiangyang	married women	6976	28.67（3.47~103.53）	[100]
2016	Nanjing	women(35-64y)	30160	99.50（67.12~141.97）	[101]
2015	Taizhou	women(35-39y)	315290	49.29（39.39~54.80）	[102]
2015	Guangzhou	women(35-64y)	1420	140.00（17.06~507.85）	[103]
2015	China	women(20-49y)	15986	56.30（25.75~106.85）	[104]
2015	Zhangjiagang	women(35-59y)	76881	20.00（12.88~35.40）	[105]
2014	Shaoxing	women	4402500	20.83（19.50~22.22）	[106]
2014	Hunan	women(35-39y)	308987	55.66（47.66~64.63）	[107]
2014	Nantong	women(35-65y)	2626	190.00（61.85~443.77）	[108]
2014	Qingyang	women(20-65y)	8000	62.50（20.30~145.79）	[109]
2014	Beijing	women(35-59y)	236749	42.20（34.37~51.37）	[110]
2014	Yulin	women	189876	5.27（2.53~9.69）	[111]
2014	Chongqing	women	90324	60.89（45.88~79.25）	[112]
2013	Nanjing	women(35-64y)	7824	50.00（13.93~130.85）	[113]
2013	Linyi	women(20-49y)	10056	50.00（16.15~116.00）	[114]
2013	Neimenggu	women(18-78y)	20000	180.00（126.10~249.11）	[115]
2013	Wuhu	women(35-69y)	10100	89.00（40.75~169.09）	[116]
2012	China	women	15413	60.00（31.12~119.28）	[117]
2012	Maanshan	women(20-65y)	21726	64.40（35.23~108.09）	[118]
2012	Jiyuan	women(25-60y)	3066	130.00（35.56~333.70）	[119]

Year of publication	Region	Age	Sample size	Prevalence rate(100000)	Reference
2012	Hangzhou	women(31-60y)	2832	30.00（0.89~196.58）	[120]
2012	Lishui	women	109537	12.00（6.32~20.29）	[121]
2012	Zunyi	women(35-69y)	5000	80.00（21.80~204.70）	[122]
2012	Wuxi	women(25-55y)	812303	10.00（7.92~12.39）	[123]
2012	Wuwei	women(30-59y)	2000	300.00（110.17~651.82）	[124]
2012	Yongjing	women(18-76y)	11892	168.18（102.76~259.62）	[125]
2012	Wuhan	women(18-49y)	58720	6.80（1.86~17.44）	[126]
2012	Guangzhou	women(18-62y)	3501	142.82（46.39~332.97）	[127]
2011	Zaozhuang	women	1987773	3.83（3.01~4.79）	[128]
2011	Zunyi	women(35-69y)	10004	90.00（47.94~183.75）	[129]
2011	Shizuishan	women	26635	15.02（4.09~38.45）	[130]
2011	Xiangxi	women	116354	10.77（5.95~19.11）	[131]
2011	Shanghai	women(≤65y)	449163	29.83（25.00~35.33）	[132]
2011	Guangzhou	women	112344	38.93（26.95~50.53）	[133]
2011	Yinchuan	women(20-49y)	35628	78.59（52.23~113.56）	[134]
2011	Luan	women(21-58y)	1389	140.00（17.44~519.16）	[135]
2011	Jiangshan	women(35-59y)	5000	80.00（21.80~204.70）	[136]

Table S12. The prevalence studies of prevalence rate of thyroid cancer in China.

Year of publication	Region	Age	Sample size	Prevalence rate(100000)	Reference
2018	Shanxi	physical examination crowd	35649	291.80（238.43~353.37）	[137]
2017	Yili	women(22-99y)	4461	650.00（435.79~932.30）	[94]
2017	Jilin	adult(20-60y)	3007	700.00（432.81~1065.56）	[138]
2014	Shaoxing	resident	4402500	20.06（18.76~21.42）	[106]
2014	China	resident	1075205128	7.80（0.20~43.45）	[139]
2011	Fujian	resident	36160000	5.45（5.21~5.70）	[140]

Table S13. The prevalence studies of prevalence rate of adult obesity in China.

Year of publication	Region	Age	Sample size	Prevalence rate(%)	Reference
2017	Jiangxi	≥15y	14964	7.9（7.5~8.4）	[141]
2019	expect Tibet	≥40y	726390	3.5（3.5~3.5）	[142]
2020	China	≥18y	174840	14.0（13.8~14.2）	[143]
2019	China	≥18y	12543	14.0（13.4~14.6）	[144]
2016	Jilin	18~79y	20839	14.6（14.1~15.1）	[145]
2020	China	≥18y	441306	5.2（5.1~5.3）	[146]
2019	Liaoning	≥40y	10891	18.5（17.8~19.2）	[147]
2018	China	≥45y	1556	16.8（15.0~18.8）	[148]
2018	China	≥45y	1556	18.3（16.4~20.3）	[148]
2018	China	≥45y	1556	16.9（15.1~18.9）	[148]
2020	Hunan	≥18y	19387	4.1（3.2~5.0）	[149]
2020	Hunan	≥18y	17959	7.1（5.1~9.2）	[149]
2016	China	20~59y	154931	12.2（12.0~12.3）	[150]
2016	China		146703	12.9（12.7~13.1）	[150]
2015	Guizhou	20~80y	2421	10.6（9.4~11.9）	[151]
2020	Shandong	≥60y	7070	16.9（16.0~17.8）	[152]
2017	Mianyang	≥18y	262434	8.8（8.7~8.9）	[153]
2013	Foshan	≥18y	26606	5.8（5.5~6.1）	[154]
2014	Beijing	≥18y	3851	19.7（18.5~21.0）	[155]
2015	Nantong	≥18y	57376	10.1（9.8~10.3）	[156]
2016	Jiaxing	≥18y	3106	8.0（7.1~9.1）	[157]
2017	Haining	≥18y	1475	6.6（5.4~8.0）	[158]
2019	Beijing	18~79y	4835	20.6（19.5~21.8）	[159]
2019	Handan	≥18y	18402	15.2（14.7~15.8）	[160]
2020	Shaoxing	18~69y	4869	16.2（15.2~17.3）	[161]

Table S14. The prevalence studies of prevalence rate of diabetes in China.

Year of publication	Region	Age	Sample size	Prevalence rate(%)	Reference
2014	China	≥40y	259657	20.9（20.7~21.0）	[162]
2017	Xian	≥18y	8150	8.0（7.5~8.6）	[163]
2020	China	≥18y	75880	12.8（12.6~13.0）	[164]
2017	China	≥18y	170287	10.9（10.8~11.0）	[165]
2013	China	≥18y	98658	11.6（11.3~11.8）	[166]
2017	Shenzhen	18~70y	1676	4.8（3.9~6.0）	[167]
2016	Shanghai	≥35y	18736	20.3（19.7~20.9）	[168]
2016	Jiangsu	≥18y	15404	7.3（6.9~7.7）	[169]
2018	Jilin	≥40y	4052	11.2（10.1~12.4）	[170]
2020	Jiangsu	35~75y	95348	19.4（19.2~19.7）	[171]
2019	Beijing	40~79y	11889	9.6（9.1~10.1）	[172]
2020	Jining	≥18y	12304	17.1（16.5~17.8）	[173]
2018	jingzhou	20~80y	5511	6.4（5.8~7.1）	[174]
2015	Tianjin	≥18y	2397	17.0（15.5~18.6）	[175]
2019	Jiangsu	≥18y	8204	11.0（10.3~11.7）	[176]
2020	Liaoning	≥40y	5424	20.2（19.1~21.3）	[177]
2017	Hubei	≥18y	5824	10.3（9.5~11.1）	[178]
2018	Gansu	20~74y	14480	9.5（9.0~10.0）	[179]
2013	Xuzhou	20~74y	2446	14.1（12.8~15.6）	[180]

Table S15. The prevalence studies of prevalence rate of male infertility in China.

Year of publication	Region	Age	Sample size	Prevalence rate(%)	Reference
2018	China	married couple(20-49y)	18571	7.7（7.4~8.1）	[181]
2016	Huludao	couple	4232	6.5（5.8~7.3）	[182]
2015	Shandong	couple	1627	6.8（5.6~8.2）	[183]
2019	Beijing	married couple(20-44y)	1053	13.8（11.7~16.0）	[184]
2015	Foshan	married couple(20-44y)	3376	1.2（0.8~1.6）	[185]
2012	Jiaxing	married couple	3175	1.4（1.0~1.8）	[186]
2012	Yinchuan	married male	1447	1.9（1.2~2.7）	[187]
2020	Dali	married couple	1386	2.9（2.1~3.9）	[188]
2017	Foshan	married couple(20-49y)	1842	7.7（6.5~9.0）	[189]

Table S16. Disease cost of intellectual disability in China.

Year of publication	Region	Age	Method	Cost per case	Reference
2013	Taiwan	≤18y	retrospective analysis	4339.4yuan/person(direct)	[190]
2013	Taiwan	≤19y	cross-section analysis	7597.4yuan/person(direct)	[191]

Table S17. Disease cost of breast cancer in China.

Year of publication	Region	Crowd	Method	Cost per case	Reference
2020	Shandong	≤60y resident	longitudinal study	9821.57yuan/person(direct)	[192]
2019	Beijing	cancer patient	retrospective analysis	2327.93yuan/person(male,direct)；2457.46yuan/person(female,direct)	[193]
2018	China	women	cross-section analysis	54661.75yuan/person(total)；48691.01yuan/person(direct)；5964.28yuan/person(indirect)	[194]
2017	Xinjiang	cancer patient	retrospective analysis	28010.3yuan/person(direct)	[195]
2016	Jinchang	cancer patient		6710.29yuan/person(direct)	[196]
2015	Henan	cancer patient		10150.78yuan/person(direct)	[197]
2014	Jinchang	cancer patient		43671.75yuan/person(direct)；16154.93yuan/person(indirect)	[198]
2014	Jinchang	cancer patient		12323.75yuan/person(direct)；3154.96yuan/person(indirect)	[199]
2013	Sichuan	cancer patient		160457yuan/person(total)	[200]
2010	Lanzhou	cancer patient		7609.78yuan/person(direct)	[201]
2010	Shanghai	cancer patient		48583.16yuan/person(direct)	[202]
2008	Shandong	cancer patient		14623.75yuan/person(direct)	[203]

Table S18. Disease cost of thyroid cancer in China.

Year of publication	Region	Crowd	Method	Cost per case	Reference
2020	Wenzhou	cancer patient		20749.95yuan/person(direct)	[204]
2019	Tianjin	cancer patient		30534.87yuan/person(direct)	[205]

Table S19. Disease cost of diabetes, adult obesity and male infertility in China.

Year of publication	Region	Crowd	Sample size	Cost per case(yuan/person)	Reference
disease	diabetes				
2013	Wuhan	diabetic	1528	1439.73^{D}	[206]
2009	Eastern China	diabetic	1668	11300.51^{D}	[207]
2010	China	diabetic	1797	11740.24^{D}	
2011		diabetic	2078	12676.38^{D}	
2010	Yunnan	village people(≥18y)	9396	$6505.13^{D}/53243.48^{I}/3286.36^{IN}$	[208]
2009	Yunnan	village people(≥35y)	6350	$6268.65^{D}/322.2^{I}$	[209]
2016		village people(≥35y)	6359	$8223.3^{D}/380.68^{I}$	
2007	China	type 2 diabetes patients	2040	$11555^{D}/1586^{I}$	[210]
2002	China	diabetic	1111	$6437.03^{D}/686.08^{I}$	[211]

Year of publication	Region	Crowd	Sample size	Cost per case(yuan/person)	Reference
2015	Nanjing, Jinzhong, Yinchuan	type 2 diabetes patients	2412	4701.2^{D}	[212]
2013	Guilin	residents	6063	$3254.83^{D}/1354.76^{I}$	[213]
2011	Kunming	village people(≥18y)	4595	$3464.49^{D}/84.48^{I}/4045.97^{IN}$	[214]
2018	Shenzhen	diabetic	120099	$1712.4^{D}/410.32^{I}$	[215]
2008	Chengdu	elderly diabetic	322	$4275.56^{D}/2265.1^{I}$	[216]
2010	Qujing	village people(≥18y)	4801	$8095.8^{D}/26.2^{I}/8370^{IN}$	[217]
2014	Sichuan	diabetic	134516	$956.55^{D}/167.12^{I}$	[218]
2011	China	residents	13830	$4558^{D}/168^{I}$	[219]
2003	Shanghai	type 2 diabetes patients	642	$3021.17^{D}/637.6^{I}$	[220]
2007	Shanghai	type 2 diabetes patients	361	$5584.45^{D}/720.77^{I}$	[221]
2016	Jiangsu	diabetic(18-65y)	2444	$3566^{D}/737^{I}/3783^{IN}$	[222]
2013	Shenzhen	residents(≥18y)	1191	1079.47^{D}	[223]
2006	Nanjing	diabetic(≥45 岁)		3079.05^{D}	[224]
2004	Shanghai	type 2 diabetes patients(40-80y)	618	$7069.61^{D}/2234.63^{I}$	[225]
2011	Tianjin	type 2 diabetes patients	250	$34724^{D}/1595^{I}$	[226]
disease	Adult obesity				
2015	Yunnan	≥ 35y	4979	$7148.55^{D}/426.43^{I}$	[227]
2000	China	/	/	$197.01^{D}/1213.71^{I}$	[228]
2000	China	≥ 18y	33530	5.30^{D}	[229]
2004			33530	33.51^{D}	
2006			33530	49.32^{D}	
2009			33530	83.62^{D}	
2010	China	≥ 18y	/	202.92^{D}	[230]
2003	China	≥ 18y	143521	78.71^{D}	[231]
disease	Male infertility				
2008	China	infertility patients	/	$20172^{D}/329.4^{I}/240^{IN}$	[232]

Note: D represents direct economic burden, I represents indirect economic burden, IN represents intangible economic burden.

References:

32. Zhao, X.; Yang, X.; Du, Y.; Li, R.; Zhou, T.; Wang, Y.; Chen, T.; Wang, D.; Shi, Z. Polybrominated diphenyl ethers in serum from residents living in a brominated flame retardant production area: Occurrence, influencing factors, and relationships with thyroid and liver function. *Environ. Pollut.* **2021**, *270*, 116046.
33. Wang, Y.; Li, M.; Ayeguli, A.; Haiqimu Khan, A.; Liu, Z. Association between polybrominated diphenyl ethers levels in umbilical cord blood and birth outcomes of neonates in Urumqi. *Prev. Med.* **2020**, *32*, 526–529.
34. Guo, J.; Miao, W.; Wu, C.; Zhang, J.; Qi, X.; Yu, H.; Chang, X.; Zhang, Y.; Zhou, Z. Umbilical cord serum PBDE concentrations and child adiposity measures at 7 years. *Ecotoxicol. Environ. Saf.* **2020**, *203*, 111009.
35. Xu, B.; Wu, M.; Wang, M.; Pan, C.; Qiu, W.; Tang, L.; Xu, G. Polybrominated diphenyl ethers (PBDEs) and hydroxylated PBDEs in human serum from Shanghai, China: A study on their presence and correlations. *Environ. Sci. Pollut. Res. Int.* **2018**, *25*, 3518–3526.
36. Wang, Y.; Liu, S.; Zhao, H.; Zhao, G.; Chen, J.; Zhai, G.; Zhao, H. Polybrominated diphenylethers (PBDEs) and their hydroxylated metabolites (OH-PBDEs) in female serum from Dalian, China. *Int. J. Hyg. Environ. Health* **2016**, *219*, 816–822.
37. Yang, L.; Lu, Y.; Wang, L.; Chang, F.; Zhang, J.; Liu, Y. Levels and Profiles of Polybrominated Diphenyl Ethers in Breast Milk During Different Nursing Durations. *Bull. Environ. Contam. Toxicol.* **2016**, *97*, 510–516.
38. Lv, Q.X.; Wang, W.; Li, X.H.; Yu, L.; Zhang, Y.; Tian, Y. Polychlorinated biphenyls and polybrominated biphenyl ethers in adipose tissue and matched serum from an E-waste recycling area (Wenling, China). *Environ. Pollut.* **2015**, *199*, 219–226.
39. Huang, F.; Wen, S.; Li, J.; Zhong, Y.; Zhao, Y.; Wu, Y. The human body burden of polybrominated diphenyl ethers and their relationships with thyroid hormones in the general population in Northern China. *Sci. Total Environ.* **2014**, *466–467*, 609–615.
40. Chen, Z.J.; Liu, H.Y.; Cheng, Z.; Man, Y.B.; Zhang, K.S.; Wei, W.; Du, J.; Wong, M.H.; Wang, H.S. Polybrominated diphenyl ethers (PBDEs) in human samples of mother-newborn pairs in South China and their placental transfer characteristics. *Environ. Int.* **2014**, *73*, 77–84.
41. Shi, Z.; Jiao, Y.; Hu, Y.; Sun, Z.; Zhou, X.; Feng, J.; Li, J.; Wu, Y. Levels of tetrabromobisphenol A, hexabromocyclododecanes and polybrominated diphenyl ethers in human milk from the general population in Beijing, China. *Sci. Total Environ.* **2013**, *452–453*, 10–18.
42. Wang, H.S.; Jiang, G.M.; Chen, Z.J.; Du, J.; Man, Y.B.; Giesy, J.P.; Wong, C.K.; Wong, M.H. Concentrations and congener profiles of polybrominated diphenyl ethers (PBDEs) in blood plasma from Hong Kong: Implications for sources and exposure route. *J. Hazard. Mater.* **2013**, *261*, 253–259.
43. Chao, H.R.; Tsou, T.C.; Huang, H.L.; Chang-Chien, G.P. Levels of breast milk PBDEs from southern Taiwan and their potential impact on neurodevelopment. *Pediatr. Res.* **2011**, *70*, 596–600.
44. Wu, K.; Xu, X.; Liu, J.; Guo, Y.; Li, Y.; Huo, X. Polyhrominated Diphenyl Ethers in Umbilical Cord Blood and Relevant Factors in Neonates from Guiyu, China. *Environ. Sci. Technol.* **2010**, *44*, 813–819.
45. Chen, G.; Huang, W.; Li, H.; Huang, W. Phthalate exposure during pregnancy and its relationship with birth outcomes in Guangzhou. *Chin. J. Environ. Occup. Med.* **2021**, *38*, 573–579.
46. Qiu, J.; Wu, H.; Mao, B.; Tang, C.; Chen, B. Correlation between phthalates exposure and neurobehavioral development in infants from a community of Shanghai. *J. Environ. Occup. Med.* **2019**, *36*, 300–305.
47. Sun, X. Associations of Prenatal Exposure to Phthalates with Fetal Hormones and Early Childhood Growth. Ph.D Thesis, Huazhong University of Science and Technology, Wuhan, China, 2019.
48. Lei, C.; Zheng, D.; Mou, L.; Cheng, Y.; Tan, Z.; Dai, X.; Zhong, H. Urinary phthalate metabolites in children aged 5–6 years in some rural areas of Chongqing. *J. Environ. Health* **2019**, *36*, 520–522.
49. Zhang, Y. Effect of Phthalate Exposure during Three Gestation Period on Birth Weight and Gender-Dependent Manner: A Birth Cohort Study in China. Ph.D Thesis, Anhui Medical University, Hefei, China, 2017.
50. Chen, C.; Wang, X.; Wang, Z.; Guo, J.; Zhao, Y.; Dong, J.; Sun, X.; Shi, Y.; Wang, Y. Analysis of phthalates exposure in college students. *J. Environ. Health* **2016**, *33*, 335–338.
51. Gao, C.; Liu, L.; Ma, W.; Zhu, N.; Liu, L.; Li, Y. Residue and health risk assessment of phthalate metabolites in urine of young adults in Northeast China. *J. Nat. Sci. Heilongjiang Univ.* **2015**, *32*, 654–660.

52. Wu, W. Study of Phthalate Exposure and Impacts on Children's Growth and Development. Ph.D. Thesis, Huazhong University of Science and Technology, Wuhan, China, 2015.
53. Wang, H. Impact of Exposure to Phthalates and Bisphenol A on Growth and Development of School Children. Ph.D. Thesis, Fudan University, Shanghai, China, 2013.
54. Li, L.; Tian, X.; Zhang, X.; Ha, L. Blood Level of PAEs in Child-bearing Period Women. *J. Environ. Health* **2011**, *28*, 792–794.
55. Miao, H.; Zhang, L.; Lai, J.; Sun, L.; Zhao, Y.; Li, J. A study on the correlation of phthalate metabolites in umbilical cord blood of 161 newborns with birth indicators in Beijing. *Chin. J. Prev. Med.* **2020**, *54*, 768–773.
56. Sheng, Y. Phthalate levels pregnant women serum and risk of hypertensive disorder complicating pregnancy. Ph.D. Thesis, Guangxi Medical University, Nanning, China, 2019.
57. Yaghjyan, L.; Sites, S.; Ruan, Y.; Chang, S.H. Associations of urinary phthalates with body mass index, waist circumference and serum lipids among females: National Health and Nutrition Examination Survey 1999–2004. *Int. J. Obes.* **2015**, *39*, 994–1000.
58. Li, Y.L.; Lv, J.; Du, Z.P.; Feng, S.; Sheng, J.; Jin, Z.X.; Liu, K.Y.; Gao, H.; Li, X.D.; Cao, H.J.; et al. The levels of phthalate exposure and associations with obesity in an elderly population in China. *Ecotoxicol. Environ. Saf.* **2020**, *201*, 110749.
59. Hou, J.W.; Lin, C.L.; Tsai, Y.A.; Chang, C.H.; Liao, K.W.; Yu, C.J.; Yang, W.; Lee, M.J.; Huang, P.C.; Sun, C.W.; et al. The effects of phthalate and nonylphenol exposure on body size and secondary sexual characteristics during puberty. *Int. J. Hyg. Environ. Health* **2015**, *218*, 603–615.
60. Buser, M.C.; Murray, H.E.; Scinicariello, F. Age and sex differences in childhood and adulthood obesity association with phthalates: Analyses of NHANES 2007–2010. *Int. J. Hyg. Environ. Health* **2014**, *217*, 687–694.
61. Zhang, Y.; Meng, X.; Chen, L.; Li, D.; Zhao, L.; Zhao, Y.; Li, L.; Shi, H. Age and sex-specific relationships between phthalate exposures and obesity in Chinese children at puberty. *PLoS ONE* **2014**, *9*, e104852.
62. Dong, R.; Zhao, S.; Zhang, H.; Chen, J.; Zhang, M.; Wang, M.; Wu, M.; Li, S.; Chen, B. Sex Differences in the Association of Urinary Concentrations of Phthalates Metabolites with Self-Reported Diabetes and Cardiovascular Diseases in Shanghai Adults. *Int. J. Environ. Res. Public Heal.* **2017**, *14*, 598.
63. Huang, T.; Saxena, A.R.; Isganaitis, E.; James-Todd, T. Gender and racial/ethnic differences in the associations of urinary phthalate metabolites with markers of diabetes risk: National Health and Nutrition Examination Survey 2001–2008. *Environ. Health* **2014**, *13*, 6.
64. Duan, Y.; Sun, H.; Han, L.; Chen, L. Association between phthalate exposure and glycosylated hemoglobin, fasting glucose, and type 2 diabetes mellitus: A case-control study in China. *Sci. Total Environ.* **2019**, *670*, 41–49.
65. Lind, P.M.; Zethelius, B.; Lind, L. Circulating levels of phthalate metabolites are associated with prevalent diabetes in the elderly. *Diabetes Care* **2012**, *35*, 1519–1524.
66. Han, X.; Cui, Z.; Zhou, N.; Ma, M.; Li, L.; Li, Y.; Lin, H.; Ao, L.; Shu, W.; Liu, J.; et al. Urinary phthalate metabolites and male reproductive function parameters in Chongqing general population, China. *Int. J. Hyg. Environ. Health* **2014**, *217*, 271–278.
67. Hauser, R.; Meeker, J.D.; Duty, S.; Silva, M.J.; Calafat, A.M. Altered semen quality in relation to urinary concentrations of phthalate monoester and oxidative metabolites. *Epidemiology* **2006**, *17*, 682–691.
68. Wang, Y.X.; You, L.; Zeng, Q.; Sun, Y.; Huang, Y.H.; Wang, C.; Wang, P.; Cao, W.C.; Yang, P.; Li, Y.F.; et al. Phthalate exposure and human semen quality: Results from an infertility clinic in China. *Environ. Res.* **2015**, *142*, 1–9.
69. Liu, L.; Bao, H.; Liu, F.; Zhang, J.; Shen, H. Phthalates exposure of Chinese reproductive age couples and its effect on male semen quality, a primary study. *Environ. Int.* **2012**, *42*, 78–83.
70. Liu, X.; Zhou, X.; Chen, W.; Jia, F.; Zhang, M.; Chen, J.; Wu, M.; Chen, B.; Li, S. Association between exposure to phthalates and type 2 diabetes in middle and old age. *Shanghai J. Prev. Med.* **2019**, *31*, 443–447.
71. Chen, Y.; Miao, M.; Liang, H.; Chen, Y.; Ji, H.; Ren, Y.; Liu, X.; Zhou, Y.; Wang, Z.; Yuan, W. Effects of prenatal exposure to polybrominated diphenyl ethers (PBDEs) on the second to fourth digit ratio in children aged 4 years. *Int. J. Hyg. Environ. Health* **2021**, *231*, 113639.
72. Pan, C.; Lu, Q.; Yao, Q.; Tian, Y.; Gao, Y. Polybrominated diphenyl ethers exposure in late pregnancy and child growth at 8 years of age. *Chin. J. Environ. Occup. Med.* **2020**, *37*, 1042–1049.

73. Fu, Z.; Sun, G.; Sun, W.; Liu, L.; Li, W.; Li, Q.; Wang, D. Exposure levels and correlation of polybrominated diphenyl ethers in serum, hair, and nail of workers from a deca-brominated diphenyl ether manufacturing plant. *J. Environ. Occup. Med.* **2020**, *37*, 579–585.
74. Luan, M.; Liang, H.; Yang, F.; Yuan, W.; Chen, A.; Liu, X.; Ji, H.; Wen, S.; Miao, M. Prenatal polybrominated diphenyl ethers exposure and anogenital distance in boys from a Shanghai birth cohort. *Int. J. Hyg. Environ. Health* **2019**, *222*, 513–523.
75. Luan, M.; Liang, H.; Wang, Z.; Ji, H.; Liu, X.; Liu, X.; Yuan, W.; Miao, M. Concentrations and influencing factors of polybrominated diphenyl ethers in cord blood of newborns in Minhang District of Shanghai. *J. Environ. Occup. Med.* **2019**, *36*, 1–10.
76. Ji, H.; Liang, H.; Wang, Z.; Miao, M.; Wang, X.; Zhang, X.; Wen, S.; Chen, A.; Sun, X.; Yuan, W. Associations of prenatal exposures to low levels of Polybrominated Diphenyl Ether (PBDE) with thyroid hormones in cord plasma and neurobehavioral development in children at 2 and 4 years. *Environ. Int.* **2019**, *131*, 105010.
77. Liu, X. The Prospective Nested Case-Control Study of the Association between Persistent Organic Pollutants Exposure and Gestational Diabetes Risk. Ph.D. Thesis, Nanchang University, Nanchang, China, 2019.
78. Lin, M. Study on the Concentration and Trend of Persistent Organic Halogenated Compounds in Serum of Weifang City and Yantai City. Ph.D. Thesis, Minzu University of China, Beijing, China, 2019.
79. Song, Q.; He, X.; Si, J.; Jin, Y.; Chen, S.; Zhang, Y. Prenatal exposure to polybrominated diphenyl ethers and intrauterine growth retardation in newborns: A nested case-control study. *J. Environ. Occup. Med.* **2018**, *35*, 209–217.
80. Cui, C.; Tian, Y.; Zhang, L.; Gao, Y.; Jin, J.; Wang, P.; Ding, W.; Wang, X.; Shi, R.; Wang, Y. Polybrominated diphenyl ethers exposure in breast milk in Shanghai, China: Levels, influencing factors and potential health risk for infants. *Sci. Total Environ.* **2012**, *433*, 331–335.
81. Ding, W. Preliminary Study on Human Exposure to Brominated Flame Retardants in Typical Areas. Ph.D. Thesis, Minzu University of China, Beijing, China, 2011.
82. He, P.; Chen, G.; Zheng, X. Prevalence of intellectual disability and concurrent impairments among Chinese children, 1987–2006. *Chin. J. Public Health* **2017**, *33*, 1417–1421.
83. Zhang, X.; Wang, P.; Wang, Y. Current status of children aged 0~6 years old from Yangzhou city in 2007. *Matern. Child Health Care China* **2014**, *29*, 424–427.
84. Wang, X. Investigation in the Status of Women Disabilities in Henan Province in 2006. Ph.D. Thesis, Zhengzhou University, Zhengzhou, China, 2012.
85. Kwok, H.W.; Cui, Y.; Li, J. Perspectives of intellectual disability in the People's Republic of China: Epidemiology, policy, services for children and adults. *Curr. Opin. Psychiatry* **2011**, *24*, 408–412.
86. Yin, H.; Huang, X. Analysis on Intellectual Disability Prevalence and Rehabilitation Demands in Zhejiang Province. *Zhejiang Prev. Med.* **2011**, *23*, 1–4.
87. Chen, R.; Chen, G.; Song, X.; Zhang, L.; Zheng, X. Epidemiological study on disabilities among ethnic minorities in China. *Chin. J. Epidemiol.* **2010**, *31*, 538–543.
88. Zheng, R.; Liang, F.; Bai, J.; Hu, Y.; Bai, Q.; Pan, L.; Li, Z. Women's diseases in Lanzhou, 2014–2018. *Mod. Prev. Med.* **2019**, *46*, 3897–3901.
89. Zhang, M.; Qiao, C.; Huang, Q.; Tang, M.; Liu, X. Prevalence of common gynecological diseases in Pudong New Area from 2011 to 2017. *J. Reprod. Med.* **2019**, *28*, 1088–1091.
90. Zhang, J. Analysis of screening results of breast and cervical benign and malignant lesions in women of Dalian from 2016 to 2017. *Med. J. Chin. People's Health* **2019**, *31*, 123–124+130.
91. Gu, L.; Ma, X. Analysis on prevalence of gynecological diseases from 2009 to 2018 in Fengxian district, Shanghai. *Nurs. Integr. Tradit. Chin. West. Med.* **2019**, *5*, 19–21.
92. Sun, X.; Jiang, Y.; Liu, H.; Zhang, X.; Huo, Y.; Han, P.; Sun, L.; Cao, W. Investigation of female breast disease in Zhengzhou. *J. Pract. Med.* **2017**, *33*, 2045–2048.
93. Ren, C. The Screening of Cervical Cancer and Breast Cancer in Four Counties of Yibin City, 2013–2015. *J. Occup. Health Damage* **2017**, *32*, 94–97.
94. Geng, X.; Li, S.; Li, S.; Wang, X.; Zhang, B.; Huang, X. Investigation on the prevalence of breast disease, gynecological disease and thyroid disease among women in Xinjiang and Buxer Country. *Women's Health Res.* **2017**, 171–172.
95. Deng, S.; Liu, C. Investigation on the situation of gynecological diseases among the community women in Weihai city. *Chin. J. Women Child. Health* **2017**, *8*, 83–86.
96. Zheng, R.; Zeng, H.; Zhang, S.; Chen, T.; Chen, W. National estimates of cancer prevalence in China, 2011. *Cancer Lett.* **2016**, *370*, 33–38.

97. Si, Q. Analysis of cervical cancer and breast cancer in Dongsheng District from 2010 to 2014. *Women's Health Res.* **2016**, 232+240.
98. Shao, Y.; Yin, G.; Xia, S. Analysis of breast cancer screening of 25,054 rural women of Yizheng in Jiangsu Province. *J. Clin. Med. Pract.* **2016**, *20*, 190–192.
99. Qu, X. Analysis of cervical and breast cancer screening results in 81,642 women. *Guide China Med.* **2016**, *14*, 167–168.
100. Huang, G.; Chen, X.; Zhang, X. Analysis of breast diseases in Xiangyang city. *J. Xiangyang Vocat. Tech. Coll.* **2016**, *15*, 33–34.
101. Hu, M. Results of cervical and breast cancer screening in Qinhuai District, Nanjing, 2013–2015. *Jiangsu J. Prev. Med.* **2016**, *27*, 244–245.
102. Zhou, D.; Xu, J.; Yang, L. Cervical cancer and breast cancer screening results among three hundred thousand rural women in Taizhou city. *Chin. Rural. Health Serv. Adm.* **2015**, *35*, 640–642.
103. Zhi, M. The prevalence of breast cancer in rural women aged 35-64 years and the role of early intervention in community. *Guide China Med.* **2015**, *13*, 45–46.
104. Zhang, L. Epidemiological Survey of Breast Diseases among Women of Childbearing Age in 25 Countries/Districts of 6 Province in China. Ph.D. Thesis, Peking Union Medical College, Beijing, China, 2015.
105. Gong, J. Analysis of cervical cancer and breast cancer screening results among 76,881 rural women in Zhangjiagang city. *Matern. Child Health Care China* **2015**, *30*, 4962–4963.
106. Zhou, W.; Fang, Y.; Ma, Y. Incidence of malignant tumor in Shaoxing of Zhejiang Province: 2012 report. *Chin. J. Health Manag.* **2014**, *8*, 110–115.
107. Tan, L.; Gao, W.; Ren, S.; Liu, J. Analysis of 308,987 cases of breast cancer screening in Hunan Province. *Matern. Child Health Care China* **2014**, *29*, 510–512.
108. Sun, X. Analysis of screening results of two cancers in 2626 women in Pingchao area. *Qingdao Med. J.* **2014**, *46*, 457–458.
109. Liang, H.; Zhang, Z. Investigation and analysis of breast diseases in 8000 rural women in Qingyang City. *Health Vocat. Educ.* **2014**, *32*, 110–112.
110. Li, M.; Han, L.; Gao, Q. Screening results of breast cancer in women of childbearing age in Beijing in 2011. *Chin. J. Women Child Health Res.* **2014**, *25*, 40–42.
111. Gong, X.; Liang, H.; Qin, Q.; Liang, T.; Li, H.; Zhang, N. Analysis of female disease census in Yulin in 2010. *Matern. Child Health Care China* **2014**, *29*, 359–361.
112. Chen, H.; Li, X.; Liao, Y.; Huang, J. Analysis of women's disease census in Yuzhong District, Chongqing from 2008 to 2013. *Chongqing Med.* **2014**, *43*, 2643–2644.
113. Zhao, R. Analysis of 7824 cases of breast diseases in Pukou District of Nanjing. *Chin. J. Women Child. Health* **2013**, *4*, 59+62.
114. Xu, J.; Wang, A. Investigation and study on distribution of breast diseases in women of childbearing age in Linyi city. *Matern. Child Health Care China* **2013**, *28*, 4204–4207.
115. Li, B.; Geng, G.; Wang, H.; Tian, C. The analysis on prevalence rate and pathogenic factors of mammary gland disease of 20,000 healthy check-up women in inner Mongolia. *J. Dis. Monit. Control.* **2013**, *7*, 397–398.
116. Bao, H.; Meng, W.; Zhu, J. Analysis of breast cancer screening results among women aged 35–69 years in Jinghu District. *Matern. Child Health Care China* **2013**, *28*, 3087–3090.
117. Zhang, M.; Huang, Z.; Zheng, Y. Estimates and prediction on incidence, mortality and prevalence of breast cancer in China, 2008. *Chin. J. Epidemiol.* **2012**, *33*, 1049–1051.
118. Yin, X. Analysis of "two cancers" screening in women of age in Ma'anshan city. *J. Anhui Health Vocat. Tech. Coll.* **2012**, *11*, 105–106.
119. Yang, G. Analysis of breast disease survey results of 3066 women in Jiyuan City in 2011. *Chin. J. Mod. Drug Appl.* **2012**, *6*, 134–135.
120. Xu, L.; Zhong, Q.; Xu, W. Comparative analysis of breast examination results of 2832 urban and rural women in Fuyang City. *Chin. Rural. Health Serv. Adm.* **2012**, *32*, 412–413.
121. Tang, Y.; Xu, M. A retrospective analysis of the general survey of women's diseases in Suichang Country for ten consecutive years. *Chin. Rural. Health Serv. Adm.* **2012**, *32*, 409–410.
122. Tan, H.; Yang, W.; Wang, C.; Cheng, X.; Kong, F.; Sun, S.; Zeng, F.; Hu, K. Report of general investigation of breast diseases among 5000 women in Huichuan district. *Matern. Child Health Care Child* **2012**, *27*, 1310–1311.
123. Sun, L.; Sheng, Z.; Gu, Y.; Zang, Y.; Yu, Q.; Bian, Y.; He, Y.; Chen, W.; Xie, M. Analysis of common gynecological diseases in Wuxi in 2011. *Matern. Child Health Care Child* **2012**, *27*, 1849–1850.

124. Ma, Y. Analysis of the methods and results of gynecological disease survey in Tianzhu Tibetan Autonomous Country. *China Health Care Nutr.* **2012**, *22*, 4074.
125. Kong, J.; Yang, R. Analysis on the incidence of breast diseases among women in Yongjing Country. *Matern. Child Health Care Child* **2012**, *27*, 793–794.
126. Ding, X.; Li, L.; Liu, W.; Jiang, Y.; Zhang, Q.; Tian, Y. Analysis of women's disease survey in Qiaokou District of Wuhan. *Chin. J. Women Child Health* **2012**, *3*, 176–178.
127. Xu, P.; Gui, Y.; Li, J.; Guo, L.; Wu, J.; Chen, J. Analysis of 3501 cases of breast disease screening. *Natl. Med. Front. China* **2012**, *7*, 89+59.
128. Wang, M. Analysis on the examination and treatment of common diseases among women in Zaozhuang City from 2006 to 2010. *Chin. Community Dr.* **2011**, *13*, 339–340.
129. Wang, C. Screening results of breast cancer among 10,004 women in Zunyi city in 2009. Ph.D. Thesis, Zunyi Medical University, Zunyi, China, 2011.
130. Li, Y.; Li, Y. Analysis on the results of gynecological disease survey in Huinong District from 2005 to 2010. *Chin. Community Dr.* **2011**, *13*, 333–334.
131. Du, J.; Zhou, C.; Zeng, L.; Wang, W.; Luo, X.; Xiao, Z.; Wang, H. Analysis on the General Survey Result of Gynecological Disease in Tujia-Miao Autonomous Prefecture from 2006 to 2010 Xiangxi. *Chin. Prim. Health Care* **2011**, *25*, 40–41.
132. Cui, J.; Lv, H. Survey results on gynecological malignant tumors in Pudong New Area of Shanghai from 2004 to 2009. *Shanghai J. Prev. Med.* **2011**, *23*, 116–118.
133. Zhu, X.; Xie, J. Analysis of 112,344 cases of gynecological diseases in Haizhu District from 2001 to 2010. *Chin. Community Dr.* **2011**, *13*, 344–346.
134. Wang, X.; Jin, T. Analysis of breast diseases in 35,628 women of childbearing age. *Nei Mong. J. Tradit. Chin. Med.* **2012**, *31*, 58–59.
135. Zhu, Q. Analysis of breast screening results of 1389 women. *Chin. Rural. Health Serv. Adm.* **2011**, *31*, 421–422.
136. Chai, Q. Breast cancer screening of rural women in Jiangshan city. *Zhejiang Prev. Med.* **2011**, *23*, 66–67.
137. Gong, J.; Zhou, J. Prevalence and incidence of malignant tumors in physical examination population in Shanxi Province from 2012 to 2015. *J. Huzhou Univ.* **2018**, *40*, 44–49.
138. Chen, H.; Zhan, D.; Feng, B.; Li, W.; Liu, T.; Zhai, J.; Wang, X.; Sun, H.; Luo, Y. Epidemiological study on adult thyroid disease in Jilin Province. *Chin. J. Ctrl. Endem. Dis.* **2017**, *32*, 241–244.
139. Chen, Y.; Guo, L.; Zhang, Y.; Shi, J.; Ren, J.; Huang, H.; Dai, M.; Li, N. Estimation and prediction on incidence, mortality and prevalence of thyroid cancer in China, 2008. *Chin. J. Dis. Control Prev.* **2014**, *18*, 200–203.
140. Xue, L.; Lai, Y.; Li, W. Analysis on characteristics of cancer prevalence in Fujian province in 2009. *J. Trop. Med.* **2011**, *11*, 1421–1424.
141. Hu, L.; Huang, X.; You, C.; Li, J.; Hong, K.; Li, P.; Wu, Y.; Wu, Q.; Wang, Z.; Gao, R.; et al. Prevalence of overweight, obesity, abdominal obesity and obesity-related risk factors in southern China. *PLoS ONE* **2017**, *12*, e0183934.
142. Tong, X.; Wang, X.; Wang, D.; Chen, D.; Qi, D.; Zhang, H.; Wang, Z.; Lu, Z.; Li, W. Prevalence and ethnic pattern of overweight and obesity among middle-aged and elderly adults in China. *Eur. J. Prev. Cardiol.* **2019**, *26*, 1785–1789.
143. Zhang, X.; Zhang, M.; Zhao, Z.; Huang, Z.; Deng, Q.; Li, Y.; Pan, A.; Li, C.; Chen, Z.; Zhou, M.; et al. Geographic Variation in Prevalence of Adult Obesity in China: Results From the 2013–2014 National Chronic Disease and Risk Factor Surveillance. *Ann. Intern. Med.* **2020**, *172*, 291–293.
144. Chen, Y.; Peng, Q.; Yang, Y.; Zheng, S.; Wang, Y.; Lu, W. The prevalence and increasing trends of overweight, general obesity, and abdominal obesity among Chinese adults: A repeated cross-sectional study. *BMC Public Health* **2019**, *19*, 1293.
145. Wang, R.; Zhang, P.; Gao, C.; Li, Z.; Lv, X.; Song, Y.; Yu, Y.; Li, B. Prevalence of overweight and obesity and some associated factors among adult residents of northeast China: A cross-sectional study. *BMJ Open* **2016**, *6*, e010828.
146. Zhang, L.; Wang, Z.; Wang, X.; Chen, Z.; Shao, L.; Tian, Y.; Zheng, C.; Li, S.; Zhu, M.; Gao, R. Prevalence of overweight and obesity in China: Results from a cross-sectional study of 441 thousand adults, 2012–2015. *Obes. Res. Clin. Pract.* **2020**, *14*, 119–126.
147. Yu, S.; Xing, L.; Du, Z.; Tian, Y.; Jing, L.; Yan, H.; Lin, M.; Zhang, B.; Liu, S.; Pan, Y.; et al. Prevalence of Obesity and Associated Risk Factors and Cardiometabolic Comorbidities in Rural Northeast China. *Biomed. Res. Int.* **2019**, *2019*, 6509083.

148. Luo, H.; Li, J.; Zhang, Q.; Cao, P.; Ren, X.; Fang, A.; Liao, H.; Liu, L. Obesity and the onset of depressive symptoms among middle-aged and older adults in China: Evidence from the CHARLS. *BMC Public Health* **2018**, *18*, 909.
149. Hua, J.; Zhang, L.; Gao, D.; Huang, Y.; Ning, P.; Cheng, P.; Li, Y.; Hu, G. Prevalence of Overweight and Obesity among People Aged 18 Years and Over between 2013 and 2018 in Hunan, China. *Int. J. Environ. Res. Public Health* **2020**, *17*, 4048.
150. Tian, Y.; Jiang, C.; Wang, M.; Cai, R.; Zhang, Y.; He, Z.; Wang, H.; Wu, D.; Wang, F.; Liu, X.; et al. BMI, leisure-time physical activity, and physical fitness in adults in China: Results from a series of national surveys, 2000–2014. *Lancet Diabetes Endocrinol.* **2016**, *4*, 487–497.
151. Wang, K.; Wang, D.; Pan, L.; Yu, Y.; Dong, F.; Li, L.; Wang, L.; Liu, T.; Zeng, X.; Sun, L.; et al. Prevalence of Obesity and Related Factors among Bouyei and Han Peoples in Guizhou Province, Southwest China. *PLoS ONE* **2015**, *10*, e0129230.
152. Hu, F.; Xu, L.; Zhou, J.; Zhang, J.; Gao, Z.; Hong, Z. Association between Overweight, Obesity and the Prevalence of Multimorbidity among the Elderly: Evidence from a Cross-Sectional Analysis in Shandong, China. *Int. J. Environ. Res. Public Health* **2020**, *17*, 8355.
153. Liu, T.; Zhou, X.; Li, W.; Peng, Y.; Liu, X.; Wang, J.; Ren, T.; Wang, L.; Yuan, P. Prevalence of Overweight and Obesity in Adult Population in Mianyang, Sichuan Province. *J. Sichuan Univ.* **2017**, *48*, 946–948+959.
154. Xie, D.; Chen, W. Epidemiological analysis of overweight and obesity among adults in Nanhai District, Foshan City, 2010. *South China J. Prev. Med.* **2013**, *39*, 59–61+64.
155. Liu, X.; An, X.; Shi, P. Relationship between overweight and obese with common chronic diseases in Shijingshan district of Beijing. *Chronic. Pathemathol. J.* **2014**, *15*, 48–51.
156. Xu, H.; Cai, B.; Huang, C.; Wang, H.; Sun, F.; Zheng, H.; Lu, C. Investigation of common chronic diseases for adults in Nantong City. *Mod. Prev. Med.* **2015**, *42*, 457–460.
157. Chen, Q.; Hua, C.; Zhou, B.; Wang, F.; Xu, X. Analysis on prevalence of overweight and obesity and their relation with diabetes, hypertension, dyslipidemia among adults in Pinghu City. *Shanghai J. Prev. Med.* **2016**, *28*, 361–365.
158. Su, Y. Epidemiological characteristics of obesity and relationship of body mass index of obesity and waist circumference with dyslipidemia among rural adults in Haining. *Chin. J. Health Lab. Tec.* **2017**, *27*, 2389–2392.
159. Deng, Y.; Liu, X.; He, Y.; Wang, X.; Tian, K.; Han, Q.; Wei, K. The epidemiological characteristics and related risk factors of overweight, obesity and central obesity among Fangshan District of Beijing adults in 2017. *J. Med. Pest. Control.* **2019**, *35*, 846–849.
160. Liu, W.; Wang, L.; Liu, S. Analysis of overweight and obesity epidemic among Han residents in rural area of Handan city. *Chin. J. Hosp. Stat.* **2019**, *26*, 411–414.
161. Fu, F.; Zhang, Y.; Wu, X. Prevalence of adult obesity and its correlation with hypertension in Diankou town, 2018. *Pract. Prev. Med.* **2020**, *27*, 849–852.
162. Bi, Y.; Lu, J.; Wang, W.; Mu, Y.; Zhao, J.; Liu, C.; Chen, L.; Shi, L.; Li, Q.; Wan, Q.; et al. Cohort profile: Risk evaluation of cancers in Chinese diabetic individuals: A longitudinal (REACTION) study. *J. Diabetes* **2014**, *6*, 147–157.
163. Hu, M.; Wan, Y.; Yu, L.; Yuan, J.; Ma, Y.; Hou, B.; Jiang, X.; Shang, L. Prevalence, Awareness and Associated Risk Factors of Diabetes among Adults in Xi'an, China. *Sci. Rep.* **2017**, *7*, 10472.
164. Li, Y.; Teng, D.; Shi, X.; Qin, G.; Qin, Y.; Quan, H.; Shi, B.; Sun, H.; Ba, J.; Chen, B.; et al. Prevalence of diabetes recorded in mainland China using 2018 diagnostic criteria from the American Diabetes Association: National cross sectional study. *BMJ* **2020**, *369*, m997.
165. Wang, L.; Gao, P.; Zhang, M.; Huang, Z.; Zhang, D.; Deng, Q.; Li, Y.; Zhao, Z.; Qin, X.; Jin, D.; et al. Prevalence and Ethnic Pattern of Diabetes and Prediabetes in China in 2013. *JAMA* **2017**, *317*, 2515–2523.
166. Xu, Y.; Wang, L.; He, J.; Bi, Y.; Li, M.; Wang, T.; Wang, L.; Jiang, Y.; Dai, M.; Lu, J.; et al. Prevalence and control of diabetes in Chinese adults. *JAMA* **2013**, *310*, 948–959.
167. Yan, X.; Xia, H.; Li, H.; Deng, X.; Yang, L.; Zhao, S.; Zou, J.; Luo, Y.; Cao, S. Diabetes in Shenzhen, China: Epidemiological investigation and health care challenges. *J. Glob. Health* **2017**, *7*, 011102.
168. Ruan, Y.; Yan, Q.H.; Xu, J.Y.; Yang, Q.D.; Yao, H.H.; Li, R.; Shi, Y. Epidemiology of Diabetes in Adults Aged 35 and Older from Shanghai, China. *Biomed. Environ. Sci.* **2016**, *29*, 408–416.
169. Wang, Q.; Zhang, X.; Fang, L.; Guan, Q.; Guan, L.; Li, Q. Prevalence, awareness, treatment and control of diabetes mellitus among middle-aged and elderly people in a rural Chinese population: A cross-sectional study. *PLoS ONE* **2018**, *13*, e0198343.

170. Zhang, F.L.; Xing, Y.Q.; Guo, Z.N.; Wu, Y.H.; Liu, H.Y.; Yang, Y. Prevalence and risk factors for diabetes and impaired fasting glucose in Northeast China: Results from the 2016 China National Stroke Screening Survey. *Diabetes Res. Clin. Pract.* **2018**, *144*, 302–313.
171. Du, W.; Su, J.; Cui, L.; Miao, W.; Zhou, J.; Qin, Y. Prevalence, awareness, treatment and control of diabetes among six areas in Jiangsu Province. *Chin. J. Prev. Contr. Chron. Dis.* **2020**, *28*, 893–897.
172. Fang, K.; Ma, A.; Li, H.; Dong, J.; Xie, J.; Xie, C.; Qi, K.; Zhou, Y.; Zhao, Y.; Dong, Z. Prevalence and Risk Factors of Diabetes Mellitus and Impairment Fasting Glucose among Residents Aged 40–79 Years in Beijing. *Chin. Gen. Pract.* **2019**, *22*, 1014–1020.
173. Guo, L.; Cui, P.; Qin, N.; Liu, M.; Cui, J.; Zhai, M. Prevalence and influencing factors of diabetes among residents in Rencheng district, Jining city. *J. Jining Med. Univ.* **2020**, *43*, 411–414.
174. Li, L.; Zeng, J.; Xue, J.; Yang, Q.; Zhong, W. Epidemiological characteristics of diabetes in Jingzhou, Hubei Province. *Mod. Prev. Med.* **2018**, *45*, 1553–1555.
175. Pan, Y.; Jiang, G.; Chang, G.; Li, J.; Xin, P. Analysis on prevalence and risk factors of diabetes mellitus in Tianjin. *Chin. J. Prev. Contr. Chron. Dis.* **2015**, *23*, 908–912.
176. Xie, W.; Zhang, J.; Zhu, Q.; Dai, Y. The prevalence of diabetes among adult residents in Jiangsu province. *Jiangsu J. Prev. Med.* **2019**, *30*, 522–525.
177. Zhang, B.; Tian, Y.; Jing, L.; Yan, H.; Li, S.; Shi, L.; Zhang, Y.; Gao, Z.; Xu, B.; Xing, L. Morbidity and influence factors of diabetes mellitus among urban residents (≥40 years) in Liaoning Province. *Chin. J. Prev. Contr. Chron. Dis.* **2020**, *28*, 823–827.
178. Zhang, L.; Zhang, Q.; He, T.; Li, Q.; Tang, Y.; Pan, J. Prevalence characteristics of diabetes and pre-diabetes among adult residents in Hubei province. *Chin. J. Public Health* **2017**, *33*, 1345–1349.
179. Zhang, Q.; Wang, B.; Tian, L.; Quan, J.; Jia, X.; Li, Y.; Wang, D.; Li, X.; Liu, J. The prevalence of diabetes mellitus among rural residents with different ethnicity in Gansu Province. *Chin. Prev. Med.* **2018**, *19*, 266–271.
180. Zhang, Q.; Ling, H.; Ying, C.; Zhao, M.; Wang, H.; Lv, X.; Li, W. Analyzing the morbidities and risk factors of diabetes mellitus and impaired glucose regulation among urban residents in Xuzhou. *Chin. J. Prev. Contr. Chron. Dis.* **2013**, *21*, 277–281.
181. Zhou, Z.; Zheng, D.; Wu, H.; Li, R.; Xu, S.; Kang, Y.; Cao, Y.; Chen, X.; Zhu, Y.; Xu, S.; et al. Epidemiology of infertility in China: A population-based study. *BJOG* **2018**, *125*, 432–441.
182. Cong, J.; Li, P.; Zheng, L.; Tan, J. Prevalence and Risk Factors of Infertility at a Rural Site of Northern China. *PLoS ONE* **2016**, *11*, e0155563.
183. Meng, Q.; Ren, A.; Zhang, L.; Liu, J.; Li, Z.; Yang, Y.; Li, R.; Ma, L. Incidence of infertility and risk factors of impaired fecundity among newly married couples in a Chinese population. *Reprod Biomed. Online* **2015**, *30*, 92–100.
184. Wang, M. Investigation and analysis of the prevalence and related situation of infertility in Beijing from 2016 to 2018. *Diet Health* **2019**, *6*, 279–280.
185. Yu, F.; Luo, S.; Chen, Y.; Wu, C. Investigation and analysis of prevalence and related situation of infertility in Foshan City. *Chin. J. Women Child. Health* **2015**, *6*, 49–51.
186. Cao, Y. An epidemiological survey of infertility of childbearing couple in Jiaxing city. *Mod. Chin. Dr.* **2012**, *50*, 25–27.
187. Qiao, G.; Fang, X.; Bai, T. A current status of infertility prevalence and a study on medical treatment tendency of rural city. *Guide China Med.* **2012**, *10*, 4–6.
188. Xu, A.; Zhang, R.; Zhang, L.; Zhu, L. Status investigation and analysis of influencing factors of infertility in Dali city. *Mod. Chin. Dr.* **2020**, *58*, 1–3.
189. Ou, H.; Liang, Q.; Peng, C. Status and influencing factors of infertility in married couples. *Qingdao Med. J.* **2017**, *49*, 379–381.
190. Lin, L.-P.; Kuan, C.-Y.; Hsu, S.-W.; Lee, T.-N.; Lai, C.-I.; Wu, J.-L.; Lin, J.-D. Outpatient visits and expenditures for children and adolescents diagnosed with autism spectrum disorders and co-occurring intellectual disability: An analysis of the national health insurance claims data. *Res. Autism Spectr. Disord.* **2013**, *7*, 1625–1630.
191. Chiang, P.H.; Chang, Y.C.; Lin, J.D.; Tung, H.J.; Lin, L.P.; Hsu, S.W. Healthcare utilization and expenditure analysis between individuals with intellectual disabilities and the general population in Taiwan: A population-based nationwide child and adolescent study. *Res. Dev. Disabil.* **2013**, *34*, 2485–2492.
192. Xu, J.; Nicholas, S.; Wang, J.; Yang, Y. A retrospective analysis of hospital treatment expenditures among young and middle age patients with cancer, 2013-2017 under health reform. *Int. J. Health Plan. Manag.* **2020**, *35*, 878–887.

193. Yin, X.; Xu, Y.; Man, X.; Liu, L.; Jiang, Y.; Zhao, L.; Cheng, W. Direct costs of both inpatient and outpatient care for all type cancers: The evidence from Beijing, China. *Cancer Med.* **2019**, *8*, 3250–3260.
194. Liao, X.Z.; Shi, J.F.; Liu, J.S.; Huang, H.Y.; Guo, L.W.; Zhu, X.Y.; Xiao, H.F.; Wang, L.; Bai, Y.N.; Liu, G.X.; et al. Medical and non-medical expenditure for breast cancer diagnosis and treatment in China: A multicenter cross-sectional study. *Asia Pac. J. Clin. Oncol.* **2018**, *14*, 167–178.
195. Li, X.; Fang, Y.; Wang, M.; Zhang, W. Analysis on the Disease Composition and Cost of Tumor Patients with Chemotherapy of a Hospital in 2015. *Chin. Med. Rec.* **2017**, *18*, 63–66.
196. Bai, Y.; Qu, H.; Pu, H.; Dai, M.; Cheng, N.; Li, H.; Chang, S.; Li, J.; Kang, F.; Hu, X. Cancer burden in the Jinchang cohort. *Chin. J. Epidemiol.* **2016**, *37*, 306–310.
197. Liu, J.; Du, J.; Zou, Q. Analysis of Influencing Factors of Hospitalization Exposure during Breast Cancer Surgery Patients. *Chin. Med. Rec.* **2015**, *16*, 49–51.
198. Zhang, M. Study on the Economic Burden and Cost-Effectiveness Analysis of Screening in Six Common Cancers. Ph.D. Thesis, Lanzhou University, Lanzhou, China, 2014.
199. Bao, J. Research on the Economic Burden of Six Common Cancers in Gansu Province. Ph.D. Thesis, Lanzhou University, Lanzhou, China, 2014.
200. Li, H.; Huang, Y.; Huang, R.; Li, J. Standard treatment cost of female breast cancer at different TNM stages. *Chin. J. Oncol.* **2013**, *35*, 946–950.
201. Wang, M.; Hu, X.; Zheng, S.; Kou, J.; Lu, Y.; Zhang, R.; Wan, X.; Li, X.; Yang, L.; Bai, Y. Analysis of direct medical expenses of common malignant tumors in the reproductive system of elderly women. *Chin. J. Gerontol.* **2010**, *30*, 3551–3553.
202. Lv, L. The Efficacy Analysis of the Community-based Screening Program in the Consequences and Costs for Breast Cancer Treatment. Ph.D. Thesis, Fudan University, Shanghai, China, 2010.
203. Sun, J. Estimation of the Burden of Malignant Neoplasms in Shandong Province. Ph.D. Thesis, Shandong University, Jinan, China, 2008.
204. Hu, J.; Wei, Y.; Zhou, Z. Analysis on the Effect of First Round Price Adjustment of Medical Service in Wenzhou. *Health Econ. Res.* **2020**, *37*, 17–20.
205. Hao, W.; Zhang, X.; Yu, Y.; Zhao, J.; Ge, Z.; Ding, B.; Sun, X.; Liu, H.; Wen, S.; You, J. Clinical significance and cost-benefit analysis of serum calcitonin assay in diagnosis and treatment of medullary thyroid carcinoma. *Chin. J. Otorhinolaryngol. Head Neck Surg.* **2019**, *54*, 506–509.
206. Chen, D.; Liu, S.; Tan, X.; Zhao, Q. Assessment of hospital length of stay and direct costs of type 2 diabetes in Hubei Province, China. *BMC Health Serv. Res.* **2017**, *17*, 199.
207. Huang, Y.; Vemer, P.; Zhu, J.; Postma, M.J.; Chen, W. Economic Burden in Chinese Patients with Diabetes Mellitus Using Electronic Insurance Claims Data. *PLoS ONE* **2016**, *11*, e0159297.
208. Le, C.; Lin, L.; Jun, D.; Jianhui, H.; Keying, Z.; Wenlong, C.; Ying, S.; Tao, W. The economic burden of type 2 diabetes mellitus in rural southwest China. *Int. J. Cardiol.* **2013**, *165*, 273–277.
209. Li, H.F.; Cai, L.; Golden, A.R. Short-Term Trends in Economic Burden and Catastrophic Costs of Type 2 Diabetes Mellitus in Rural Southwest China. *J. Diabetes Res.* **2019**, *2019*, 9626413.
210. Wang, W.; Fu, C.W.; Pan, C.Y.; Chen, W.; Zhan, S.; Luan, R.; Tan, A.; Liu, Z.; Xu, B. How do type 2 diabetes mellitus-related chronic complications impact direct medical cost in four major cities of urban China? *Value Health* **2009**, *12*, 923–929.
211. Chen, X.; Tang, L.; Chen, H.; Zhao, L.; Hu, S. Accessing the impact of complications on the costs of Type 2 diabetes in urban China. *Chin. J. Diabetes* **2003**, *11*, 238–241.
212. Dong, W.; Lou, Q.; Wu, L.; Wu, H.; Mao, F.; Jiang, Y.; Zhang, S.; Qi, L.; Zhang, Y.; Ma, S. Direct economic burden of type 2 diabetes mellitus and its influencing factors among community patients. *Chin. J. Public Health* **2019**, *35*, 1457–1460.
213. Guo, Z.; Shi, W.; Ma, M.; Yang, L.; Liu, Y. A study on the prevalence and economic burden of disease of hypertension and diabetes in Guilin City. *Chin. J. Dis. Control Prev.* **2015**, *19*, 887–889+893.
214. He, R.; Cai, L.; Dong, J.; Tao, J.; Zhang, S. Analysis of the prevalence and Economic Burden of Diabetes in Guandu District of Kunming. *J. Kunming Med. Univ.* **2014**, *35*, 12–14.
215. Li, Q.; Zhou, S.; Chen, P.; Wu, L. Study on the Economic Burden of Diabetic Diseases in Shenzhen. *Health Econ. Res.* **2021**, *38*, 63–66.
216. Lin, M.; Jia, X.; Chen, S. Analysis medical cost on influential factor of old age diabetic in Chengdu region. *Sichuan Med. J.* **2010**, *31*, 1014–1016.
217. Liu, G.; Cai, L.; Shu, Z.; Ye, Y.; Feng, R.; Zhao, K.; He, J.; Mao, H. Analysis of Economic Burden of diabetes in Luoping Country of Yunnan Province. *Mod. Prev. Med.* **2012**, *39*, 799–800+805.

218. Ming, X.; Yang, L.; Sun, Q.; Haung, S.; Wang, M.; Wei, H. The Disease Economic Burden and Influence of Diabetes in Sichuan Province. *Health Econ. Res.* **2019**, *36*, 29–31+37.
219. Qin, J.; Zhang, Y.; Zhang, L.; Rui, D.; Mao, L.; Wang, L.; Wu, N. Prevalence of non-communicable diseases and economic burden of patients in 8 typical cities. *Chin. J. Public Health* **2014**, *30*, 5–7.
220. Qiu, Y.; Ye, L.; Li, X.; Lu, W. Economic burden of type 2 diabetes mellitus in Shanghai. *Chin. Health Resour.* **2005**, 69–71.
221. Wang, J.; Liu, Z.; Wang, W.; Fu, C.; Xu, B. Cost of illness in outpatient visit with type 2 diabetes mellitus in Shanghai. *Chin. Health Resour.* **2008**, *3*, 127–129.
222. Wang, X.; Hu, D.; Zhu, Y.; Shi, F.; Guo, H. An Empirical Study on the Economic Burden of Diabetes in Jiangsu Province. *Chin. Prim. Health Care* **2020**, *34*, 5–9.
223. Xiang, Y.; Wei, S.; Su, J.; Song, P.; Zhao, R.; Xie, Y. Analysis on epidemiological characteristics and economic burden of chronic disease among adults in Futian District, Shenzhen. *Mod. Prev. Med.* **2015**, *42*, 3169–3171.
224. Zhang, K.; Jiang, L.; Qiu, X.; Li, C. Study on the direct economic burden of diseases about middle-aged and old patient with chronic diseases in Nanjing. *Jiangsu Prev. Med.* **2009**, *20*, 4–7.
225. Zhang, Z.; Chen, J.; Tang, Z.; Hu, R.; Lu, B. Study on direct health expenditure of diabetes mellitus in China. *Chin. Health Resour.* **2007**, 162–163+168.
226. Zheng, Y.; Wu, J.; Chen, Z.; Wang, Y.; Sun, H. Economic burden and influencing factors of insured patients with type 2 diabetes mellitus in Hedong District of Tianjin. *Chin. J. Health Stat.* **2013**, *30*, 904–906.
227. Li, Q.; Cai, L.; Cui, W.; Wang, G.; He, J.; Golden, A.R. Economic burden of obesity and four obesity-related chronic diseases in rural Yunnan Province, China. *Public Health* **2018**, *164*, 91–98.
228. Popkin, B.M.; Kim, S.; Rusev, E.R.; Du, S.; Zizza, C. Measuring the full economic costs of diet, physical activity and obesity-related chronic diseases. *Obes. Rev.* **2006**, *7*, 271–293.
229. Qin, X.; Pan, J. The Medical Cost Attributable to Obesity and Overweight in China: Estimation Based on Longitudinal Surveys. *Health Econ.* **2016**, *25*, 1291–1311.
230. Zhang, J.; Shi, X.; Liang, X. Economic costs of both overweight and obesity among Chinese urban and rural residents, in 2010. *Chin. J. Epidemiol.* **2013**, *34*, 598–600.
231. Zhao, W.; Zhai, Y.; Hu, J.; Wang, J.; Yang, Z.; Kong, L.; Chen, C. Economic burden of obesity related chronic diseases in China. *Chin. J. Epidemiol.* **2006**, 555–559.
232. Zheng, X.; Qiu, Y. Disease burden of infertility in China. *Chin. J. Public Health* **2012**, *28*, 257–260.
